# Diverse PFAS produce unique transcriptomic changes linked to developmental toxicity in zebrafish

**DOI:** 10.3389/ftox.2024.1425537

**Published:** 2024-07-22

**Authors:** Yvonne Rericha, Lindsey St. Mary, Lisa Truong, Ryan McClure, J. Kainalu Martin, Scott W. Leonard, Preethi Thunga, Michael T. Simonich, Katrina M. Waters, Jennifer A. Field, Robyn L. Tanguay

**Affiliations:** ^1^ Environmental and Molecular Toxicology Department, College of Agricultural Sciences, Oregon State University, Corvallis, OR, United States; ^2^ Sinnhuber Aquatic Research Laboratory, Oregon State University, Corvallis, OR, United States; ^3^ Pacific Northwest National Laboratory, Biological Sciences Division, Richland, WA, United States; ^4^ Biological Sciences Department, College of Sciences, North Carolina State University, Raleigh, NC, United States

**Keywords:** PFAS, developmental toxicity, transcriptomics, bioconcentration, phenotypically anchored

## Abstract

Per- and polyfluoroalkyl substances (PFAS) are a widespread and persistent class of contaminants posing significant environmental and human health concerns. Comprehensive understanding of the modes of action underlying toxicity among structurally diverse PFAS is mostly lacking. To address this need, we recently reported on our application of developing zebrafish to evaluate a large library of PFAS for developmental toxicity. In the present study, we prioritized 15 bioactive PFAS that induced significant morphological effects and performed RNA-sequencing to characterize early transcriptional responses at a single timepoint (48 h post fertilization) after early developmental exposures (8 h post fertilization). Internal concentrations of 5 of the 15 PFAS were measured from pooled whole fish samples across multiple timepoints between 24–120 h post fertilization, and additional temporal transcriptomics at several timepoints (48–96 h post fertilization) were conducted for Nafion byproduct 2. A broad range of differentially expressed gene counts were identified across the PFAS exposures. Most PFAS that elicited robust transcriptomic changes affected biological processes of the brain and nervous system development. While PFAS disrupted unique processes, we also found that similarities in some functional head groups of PFAS were associated with the disruption in expression of similar gene sets. Body burdens after early developmental exposures to select sulfonic acid PFAS, including Nafion byproduct 2, increased from the 24–96 h post fertilization sampling timepoints and were greater than those of sulfonamide PFAS of similar chain lengths. In parallel, the Nafion byproduct 2-induced transcriptional responses increased between 48 and 96 h post fertilization. PFAS characteristics based on toxicity, transcriptomic effects, and modes of action will contribute to further prioritization of PFAS structures for testing and informed hazard assessment.

## 1 Introduction

Per- and polyfluoroalkyl substances (PFAS) are a structurally diverse class of chemicals to which we are frequently exposed, with some increasingly associated with adverse health effects (Sunderland et al., 2019; Fenton et al., 2021). Despite various definitions, the OECD and United States Environmental Protection Agency (US EPA) have identified thousands of unique and structurally diverse PFAS (OECD, 2021; USEPA, 2022). The defining carbon-fluorine chemistry, and the characteristic dual hydrophobic and oleophobic properties, imparts beneficial properties like resistance to heat, weathering, and staining, and surfactant capabilities. As a result, PFAS have been used in a range of applications, including electronics, cleaning products, paper products, and textiles (Gluge et al., 2020). While PFAS are very beneficial for such applications, their chemistry also makes them highly persistent in the environment and has led to their ubiquitous detection in the environment and human populations (Abunada et al., 2020). Humans are primarily exposed to PFAS via diet and drinking water, though exposure can also occur through inhalation or dermal contact (Sunderland et al., 2019). Widespread exposure and evidence that some PFAS bioaccumulate and cause adverse health effects contribute to growing concerns regarding PFAS toxicity (Fenton et al., 2021).

Toxicological studies in recent decades have often focused on a small subset of PFAS, predominately perfluoroalkyl carboxylic acids, such as perfluorooctanoic acid (PFOA), and perfluoroalkyl sulfonic acids like perfluorooctane sulfonic acid (PFOS) (Fenton et al., 2021). PFAS have been associated with adverse immune, growth, and metabolic outcomes, including disrupted lipid profiles, diabetes, and thyroid disease in epidemiological studies (Liew et al., 2018; Sunderland et al., 2019). Experimental models have also provided evidence of endocrine disruption and developmental, hepatic, metabolic, and immunotoxic effects (Fenton et al., 2021). Some PFAS pose significant human health hazard; however, little to no toxicity data are available for most of the thousands of PFAS on the global market. Furthermore, the underlying mechanisms that lead to toxicity are not definitively understood (Fenton et al., 2021). Structurally distinct PFAS differentially induce toxicity (i.e., cause varying adverse effects and magnitudes of effects) when assessed *in vitro* and *in vivo* and disrupt different biological processes based on transcriptomic analyses (Houck et al., 2021; Truong et al., 2022; Rericha et al., 2023). PFAS also exhibit varied affinities for binding to biological targets (e.g., nuclear receptors such as peroxisome proliferator-activated receptors) (Houck et al., 2021), all of which suggest that diverse PFAS likely induce adverse effects through different mechanisms. To address these data gaps, alternative models are employed to achieve rapid evaluation of large libraries of PFAS and ‘omics level investigations.

The highly resource intensive (e.g., cost, time, laboratory space) nature of traditionally used and established *in vivo* mammalian models paired with the tremendous number of chemicals lacking sufficient toxicity data have contributed to the push towards new approaches such as *in vitro* screening (Schmeisser et al., 2023). However, *in vitro* models lack biological complexity, making it challenging to predict toxicity at the organism level (Truong et al., 2014). The zebrafish (*Danio rerio*) is an alternative model that bridges the gap between mammalian and *in vitro* models. High fecundity paired with the *ex vivo*, optically clear, and rapid development of zebrafish allow for high-throughput evaluation of chemical bioactivity (Horzmann and Freeman, 2018). The zebrafish has been increasingly employed as a model for human health to evaluate developmental toxicity of PFAS (Rericha et al., 2023), and several recent studies conducted high-content screening (Dasgupta et al., 2020; Han et al., 2021; Truong et al., 2022). We recently evaluated the developmental toxicity of a library of 139 PFAS obtained from the US EPA and identified 31 molecules that induced morphological effects, such as edema, craniofacial malformations, bent axis, mortality, and more described in Supplementary Table S1A (Truong et al., 2022). From these bioactive PFAS and additional testing of Nafion byproduct 2 (NB2), the present study prioritized 15 PFAS (Table 1) by selecting compounds that elicited high incidence of any morphological effect, approximately 80%, a threshold used in previous studies of other chemical classes (Shankar et al., 2019; Dasgupta et al., 2021; Rericha et al., 2022). These 15 PFAS were used to conduct a follow-up phenotypically anchored transcriptomic investigation. Interrogating chemical-induced transcriptional changes in gene expression can provide insight into the mechanisms by which PFAS induce toxicity, an understanding of which is essential for informed PFAS hazard assessment. The zebrafish genome is fully sequenced, well annotated, and reveals significant genetic homology with humans (Howe et al., 2013), making zebrafish an effective model for transcriptomic studies with implications for environmental and human health.

**TABLE 1:** PFAS evaluated in present study during initial transcriptomic investigation. PFAS numbering was established after completion of experiments to correspond to increasing number of differentially expressed genes up to PFAS 13. BMD_80_ was determined during developmental toxicity testing (A. in Figure 1) by Truong et al. (2022) for 14 PFAS, and exposure concentration for the initial transcriptomic investigation in this study (Methods Section 2.4) was determined through a series experiments to confirm the target effective concentration.

PFAS number	PFAS name	Common abbreviation	Stock concentration in DMSO (mM)	CAS	BMD_80_ (µM)	Exposure concentration (µM) (methods 2.4)	Chemical structure
1	9-Chloro-perfluorononanoic acid	—	30	865-79-2	81.4	100	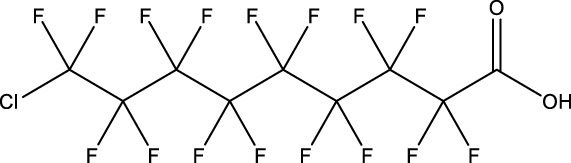
2	7H-Perfluoro-4-methyl-3,6-dioxaoctanesulfonic acid	Nafion Byproduct 2	22	7,49,836-20-2	28.2^a^	30.0	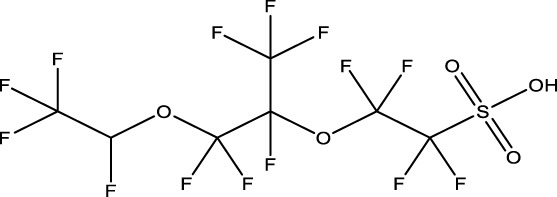
3	Potassium Perfluorooctane sulfonate	PFOS	30	2,795-39-3	13.8	13.8	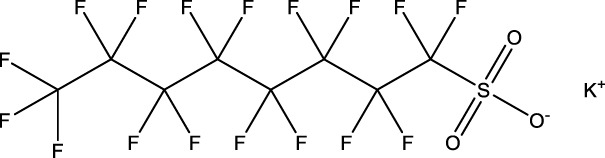
4	Perfluoro-3,6,9-trioxatridecanoic acid	—	30	3,30,562-41-9	92.2	50.0	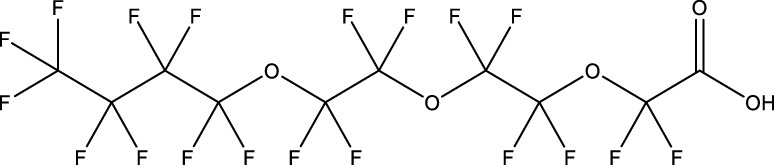
5	Perfluoroheptane sulfonic acid	PFHpS	20	375-92-8	98.7	100	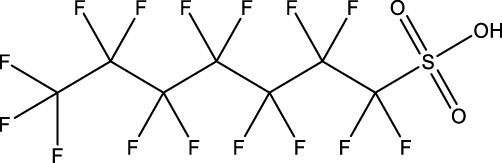
6	N-Ethyl perfluorooctane sulfonamide	EtFOSA	30	4,151-50-2	>100	100	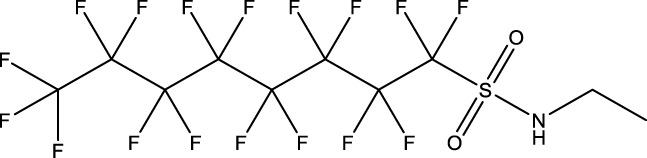
7	1-(Perfluorofluorooctyl) propane-2,3-diol	—	5	94,159-84-9	39.7	39.7	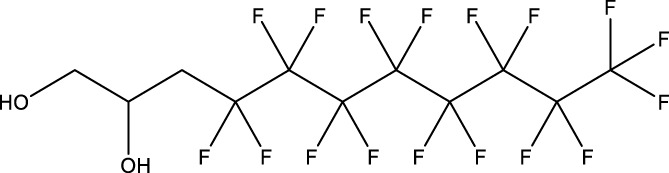
8	1H,1H,11H,11H-Perfluorotetraethylene glycol	—	30	3,30,562-44-2	28.6	53.0	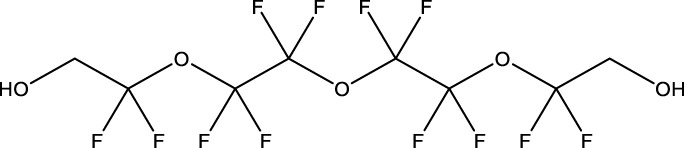
9	2,2,3,3-Tetrafluoropropyl acrylate	—	30	7,383-71-3	56.0	62.0	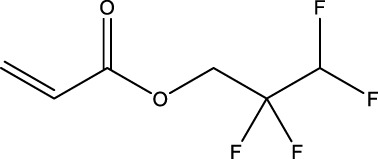
10	Perfluorooctane sulfonamide	FOSA	30	754-91-6	3.6	8.0	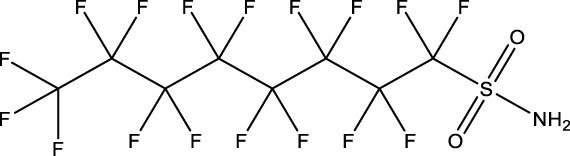
11	Perfluorohexane sulfonamide	FHxSA	30	41,997-13-1	12.5	28.0	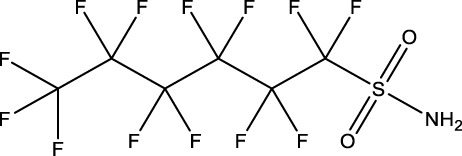
12	3H-Perfluoro-2,2,4,4-tetrahydroxypentane	—	30	77,953-71-0	41.8	100	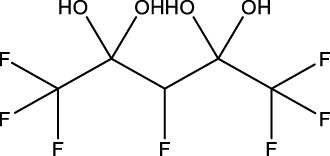
13	1H,1H,10H,10H-Perfluorodecane-1,10-diol	—	30	754–96-1	14.0	25.0	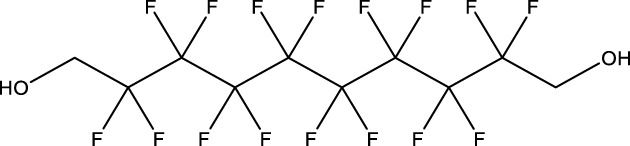
14	1H,1H,5H,5H-Perfluoro-1,5-pentanediol diacrylate	—	30	678-95-5	12.5	20.0	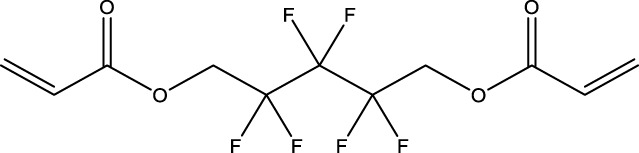
15	1H,1H,6H,6H-Perfluorohexane-1,6-diol diacrylate	—	30	2,264-01-9	15.3	20.0	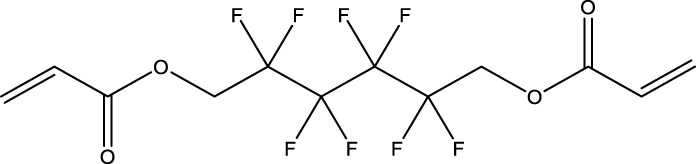

^a^ PFAS evaluated as methanol stock during initial developmental toxicity testing.

To our knowledge, RNA-sequencing has been performed in developing zebrafish for only a few PFAS, including PFOS (Martinez et al., 2019; Christou et al., 2020; Huang et al., 2021; Lee et al., 2021; Liu et al., 2022), PFOA (Liu et al., 2022; Yu et al., 2022), perfluorobutane sulfonic acid (PFBS) (Sant et al., 2019), perfluorooctane sulfonamide (FOSA) (Dasgupta et al., 2020; Chen et al., 2022; Liu et al., 2022), perfluorobutane sulfonamide (FBSA) (Rericha et al., 2022), and GenX, hexafluoropropylene oxide dimer acids (HFPO-DA), and hexafluoropropylene oxide trimer acids (HFPO-TA) (Gong et al., 2023; Sun et al., 2023), as well as NB2 (Gui et al., 2023a). Despite varied exposure paradigms and collection timepoints ranging from 14 to 120 h post fertilization (hpf), nearly all studies highlighted perturbed biological pathways related to lipid metabolism and homeostasis processes. Some authors also reported affected pathways involved in cardiotoxicity, i.e., muscle contraction and heart dysfunction (Christou et al., 2020; Chen et al., 2022; Liu et al., 2022; Yu et al., 2022), and in ion homeostasis (Rericha et al., 2022; Yu et al., 2022), immune functions (Martinez et al., 2019; Liu et al., 2022; Rericha et al., 2022), and more. While the number of ‘omics studies addressing PFAS toxicity is increasing, definitive modes of action of PFAS toxicity have not yet been established (Fenton et al., 2021). This may in part be due to the challenge of translating data between different model organisms, exposure paradigms, and life stages. Therefore, transcriptomic analysis of numerous PFAS in a single, highly standardized system will enable direct comparison between compounds and greater insight into modes of action.

**FIGURE 1:**
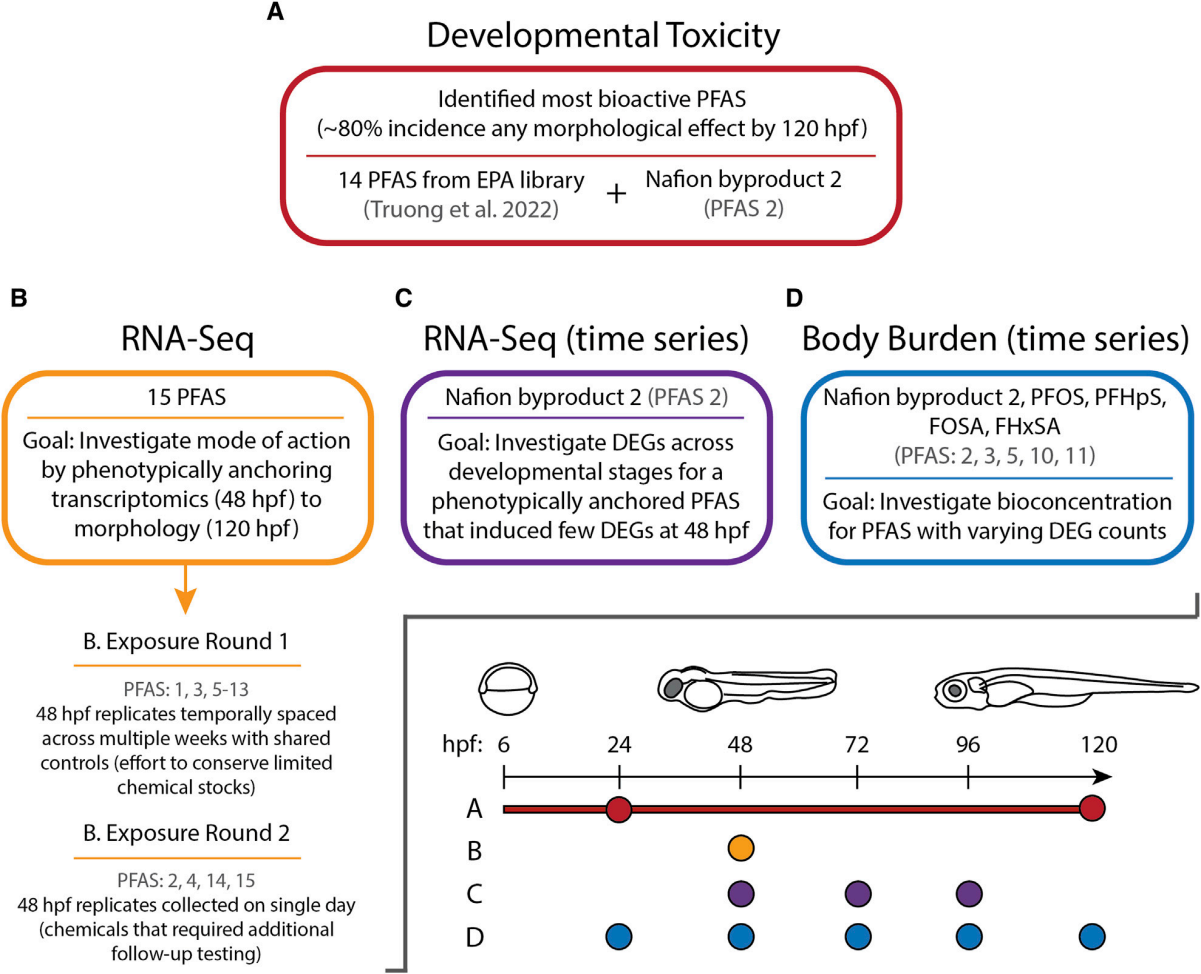
Experimental overview illustrating the sections of this study **(A–D)**, which PFAS were assessed during each section, and at which timepoints measurements were taken in hours post fertilization (hpf).

The present study characterized transcriptomic responses to 15 structurally diverse bioactive PFAS in developing zebrafish under standardized conditions previously employed to evaluate other chemical classes (Shankar et al., 2019; Dasgupta et al., 2021). Our aim was to phenotypically anchor transcriptional changes to whole-animal phenotypes. If possible, exposures were conducted at concentrations that induced approximately 80% incidence (EC_80_) of any morphological effects by 120 hpf, but zebrafish were sampled at 48 hpf prior to the onset of morphologies for transcriptome analyses. As a follow-up to these initial experiments, the bioconcentration of 5 of the 15 PFAS was measured across multiple timepoints from 24 to 120 hpf, and temporal transcriptomics for NB2 were also measured across timepoints from 48 to 96 hpf (rather than just the single timepoint measured for the other 14 PFAS above; Figure 1). In doing so, this study identified unique and commonly affected early gene expression profiles resulting from PFAS exposures that could drive adverse effects, began to elucidate mechanisms of toxicity through perturbed biological processes, and investigated the relationship between early transcriptomic changes and bioconcentration across developmental stages for a few compounds.

## 2 Materials and methods

### 2.1 Zebrafish husbandry

All protocols were approved by Oregon State University’s Institutional Animal Care and Use Committee. Tropical 5D wild-type zebrafish (*Danio rerio*) were bred and maintained at the Sinnhuber Aquatic Research Laboratory (SARL) at Oregon State University at 28°C, 1,000 fish per 100-gallon tank densities, on a 14:10 h light/dark cycle. The recirculating water system supplemented with Instant Ocean salts, health monitoring, and feeding of age-appropriate GEMMA Micro food (Skretting, Stabanger, Norway) 2–3 times per day were maintained according to protocols by Barton et al. (Barton et al., 2016). Embryo collection between 8–9 a.m., after lights turned on, was facilitated by placing a custom spawning funnel into tanks the night prior to collection. Embryos were then sorted to keep only those that were successfully fertilized, high quality, and similarly staged (Kimmel et al., 1995). Embryos were kept at 28°C in embryo medium (EM) consisting of 15 mM NaCl, 0.5 mM KCl, 1 mM MgSO_4_, 0.15 mM KH_2_PO_4_, 0.05 mM Na_2_HPO_4_, and 0.7 mM NaHCO_3_ (Westerfield, 2000) until further processing.

### 2.2 Chemicals and experiment overview

One hundred thirty-nine PFAS were previously procured from the US EPA Center for Computational Toxicology and Exposure as 5–30 mM stocks in dimethyl sulfoxide (DMSO) and evaluated for developmental toxicity in zebrafish (Truong et al., 2022). Briefly, Truong et al. exposed embryos to 0.015–100 µM PFAS, all exposure solutions normalized to 0.33% DMSO, in 96-well plates from 6 to 120 hpf, evaluating behavior and 13 morphology endpoints at 24 and 120 hpf. From this library, 13 of the most bioactive PFAS were identified for investigation in the present study, i.e., those that elicited a modeled benchmark dose corresponding to 80% incidence of any morphological effect by 120 hpf relative to background (BMD_80_) (all except PFAS 2 and 6 in Table 1, numerically labeled after the completion of the study for ease of discussion and to correspond to increasing number of differentially expressed genes observed in the present study, up to PFAS 13). Truong et al. calculated BMD_80_ using parametric curve fitting following the EPA BMDS v3.2 manual (US Environmental Protection Agency EPA, 2020). Specifically, the unrestricted 3-parameter log-logistic model for dichotomous data with “extra risk” was used (Truong et al., 2022). Stocks from the US EPA remaining from the previous study by Truong et al. were used in exposures for transcriptomics in the present study. To ensure that all solvent concentrations were identical across exposures, specifically given lower stock concentrations for some selected compounds, all exposure solutions were normalized to 1% DMSO in all subsequent testing for transcriptomics.

In addition to these 13 PFAS selected based on BMD_80_, two additional PFAS were selected for further investigation. N-ethyl perfluorooctane sulfonamide (PFAS 6) was selected from the 139 PFAS library despite a BMD_80_ greater than the highest previously tested concentration of 100 µM (Truong et al., 2022), because it exhibited high bioactivity and is structurally similar to perfluorooctane sulfonamide (FOSA; PFAS 10). NB2 (PFAS 2) was not part of the previously tested library but was selected for developmental toxicity testing and transcriptomics due to its recent detection in human serum (Kotlarz et al., 2020) and its polyfluoroether sulfonic acid structure, which added to the structural diversity of the present set of chemicals. NB2 was purchased from Synquest Laboratories (Lot: 512400, 95% purity; Alachua, FL, United States). A 22 mM stock in 100% LC/MS grade methanol was made and stored in a 15 mL polypropylene bottle. Use of methanol as the carrier solvent enabled efficient and direct analytical validation of stock concentration prior to developmental toxicity assessments across 1–100 µM concentrations, approximating the concentration range tested by Truong et al., 2022. A separate 22 mM NB2 stock in 100% DMSO was made and used in exposures for transcriptomics alongside the other 14 PFAS at a single timepoint, thus maintaining carrier solvent consistency with PFAS from the US EPA library.

Following developmental toxicity testing of NB2 and transcriptomics for the 15 PFAS, several chemicals were selected for follow-up experiments (Figure 1). Further transcriptomic investigation of NB2 across a developmental time series was conducted using a new 22 mM stock in 100% DMSO, but same source and lot listed above. Additionally, the 22 mM NB2 stock in methanol and 22 mM stocks of PFOS (PFAS 3), perfluoroheptane sulfonic acid (PFHpS; PFAS 5), FOSA (PFAS 10), and perfluorohexanesulfonamide (FHxSA; PFAS 11) in methanol were used to investigate body burden across a developmental time series. Chemical selection was based on transcriptomic findings from the present study (i.e., compounds that differed in the number of differentially expressed genes), structural homology, chemical availability, and analytical standard availability to enable quantitative internal concentration measurement.

### 2.3 Developmental toxicity assessment of Nafion byproduct 2 (NB2)

#### 2.3.1 Chemical exposures

As NB2 was a chemical of interest but not evaluated as part of the 139 PFAS from our previous study (Truong et al., 2022), a separate developmental toxicity assessment following the same protocols was conducted for NB2. At 4 hpf, embryos were enzymatically dechorionated using pronase in a custom automated system (Mandrell et al., 2012). Dechorionated embryos were placed into round-bottom 96-well plates (Falcon^®^, Product no. 353227) containing 100 μL EM by an automated placement system (Mandrell et al., 2012). Static chemical exposures were conducted following protocol established by Rericha et al., 2021, using the NB2 stock in 100% methanol to enable direct analytical measurement of the stock before use. At 6 hpf, 50 μL EM was removed from each well, followed by the addition of 50 µL working solution diluted to achieve final nominal exposure concentrations of 0, 1.0, 2.5, 6.5, 16, 35, 75, and 100 μM, 1 row per concentration across three plates (n = 36). All wells were normalized to 0.5% methanol (Rericha et al., 2021). 96-well plates were sealed (VWR, Cat no. 89134-428), placed on an orbital shaker at 235 rpm overnight, and maintained in the dark at 28°C until assessment.

#### 2.3.2 Developmental toxicity assessments

Embryos exposed to NB2 were challenged in assays previously used to test the suite of 139 PFAS (Truong et al., 2022). At 24 hpf, still maintaining darkness, embryos were challenged with an embryonic photomotor response (EPR) assay in 96-well plate format (Reif et al., 2016). The EPR assay evaluated non-visual photomotor response (i.e., tail contractions) following perception of light stimulus (1 s white light pulses) by photoreceptors within the hindbrain. Embryos were then evaluated for mortality, developmental delays (i.e., animals did not develop structures present in control animals at 24 hpf, or already possessed observable abnormal physiology), and spontaneous movement (Supplementary Tables S1A, B). Incidences of all mortality and morphology were recorded as a binary response, and assessments were blinded. After the EPR assay, 96-well plates were returned to darkness and 28°C conditions.

At 120 hpf, a larval photomotor response (LPR) assay consisting of alternating 3 min light and dark periods, in total 24 min with the first 18 min considered as acclimation, was conducted by placing the 96-well plates into ZebraBox chambers (Viewpoint Behavior Technology). ZebraLab analysis software tracked movement, and the distance traveled by each larva was integrated over 6 s time bins. After the LPR assay, mortality and nine morphological endpoints were evaluated (Supplementary Tables S1A, B).

Calculation of a BMD_80_ for NB2 was performed using the same procedure as Truong et al. to enable comparison to the other previously tested chemicals and also concentration selection for transcriptomics (Truong et al., 2022). Differences in total movement of exposed and control embryos in the EPR assay were determined using a Kolmogorov-Smirnov (K-S) test with Bonferroni correction (*p* < 0.05). Prior to statistical analysis of LPR behavior data, dead and malformed fish were removed from the datasets and 70% of the original sample size was required to proceed with analysis. For LPR, statistical differences in total distance moved were identified using area under the curve ratios and a K-S test (Zhang et al., 2017).

### 2.4 Transcriptomic investigation of 15 PFAS

#### 2.4.1 Chemical exposures

For the initial transcriptomics portion of this study, embryos underwent the same dechorionation and 96-well plate placement as described in Section 2.3 and were exposed to the selected 15 PFAS (Table 1). As the chemical stock solutions were in 100% DMSO, it was possible to use an HP D300 digital dispenser to perform the chemical exposures by dispensing stocks into individual wells containing 100 μL at 6 hpf (Truong et al., 2022). All wells were normalized to 1% DMSO. Informed by the modeled BMD_80_ values (Table 1), a series of exposures was conducted to confirm the nominal effective concentration for each chemical to elicit 80% incidence of any cumulative morphological effects (EC_80_) by 120 hpf. Confirmation testing to obtain EC values included fewer concentrations, not full concentration-response modeling, to conserve limited chemical stocks. This effective concentration threshold (80%) was chosen to ensure sufficient biological effect in the pool of larvae for phenotypical anchoring of transcriptomics and has been used in previous studies (Shankar et al., 2019; Dasgupta et al., 2021; Rericha et al., 2022). Embryos were statically exposed to the confirmed EC_80_ of each PFAS, or 100 µM if the EC_80_ were to exceed this threshold, from 6 to 48 hpf (Table 1). The 48 hpf timepoint was selected to align with previous studies of other chemicals and to precede the onset of morphological effects (Shankar et al., 2019; Dasgupta et al., 2021; Rericha et al., 2022). Given the scale of this experiment and the number of compounds being tested, only a single sampling timepoint and exposure concentration for each chemical were possible. Following chemical dispensing, plates were sealed, placed on an orbital shaker overnight at 235 rpm, and maintained in darkness at 28°C.

#### 2.4.2 Sample collection and phenotypic anchoring

Sample collection for transcriptomics was divided into two rounds. One was temporally dispersed where 48 hpf samples were collected on different days (i.e., exposures for replicate samples of all chemicals were performed on different days). The other occurred in a single day due to difficulties achieving consistent EC_80_ confirmation when attempted during the first round (Figure 1). During both rounds, 10 zebrafish embryos were pooled per replicate at 48 hpf. Pooled embryos were placed into 1.5 mL safe-lock tubes (Eppendorf, Cat no. 022363212) and euthanized on ice. Excess water was immediately removed from the tubes, 200 µL RNAzol^®^ RT (Molecular Research Center) and 100 µL 0.5 mm zirconium oxide beads (Next Advance) were added, and samples were homogenized in a Bullet Blender^®^ (Next Advance) at speed 8 for 3 min. After homogenization, another 300 µL RNAzol^®^ was added, and samples were stored at −80°C. The 48 hpf timepoint preceded morphological effects that were expected to emerge later in development. Sampling at 48 hpf provides insight into transcriptional changes that are likely nearer to molecular initiating events leading to toxicity. Additionally, as a standard practice in our laboratory, it enables transcriptomic analysis of various chemical classes at a consistent timepoint and in a standardized system (Shankar et al., 2019; Dasgupta et al., 2021; Rericha et al., 2022). To ensure phenotypic anchoring, 96-well plates with remaining embryos after sampling (i.e., hold back plates) were returned to darkness and 28°C conditions until 120 hpf when morphological effects were evaluated. Exposure Round 1 hold back plate typical sample sizes each day for controls ranged from 14 to 30 and exposed fish from 22 to 44, while Exposure Round 2 hold back plates had 40 control fish and 40–42 exposed fish.

Exposure Round 1 comprised six collection days over the span of 5 weeks, for PFAS 1, 3, and 5–13. Temporally spacing replicates allowed for conservation of limited stock solutions (i.e., multiple small exposure setups for individual replicates allowed for troubleshooting and refining of concentrations instead using large quantities of chemicals for an “all or nothing” approach), as well as the assessment of variability by day. Four replicates for all PFAS were selected for RNA-seq based on 1) achievement of target % incidence effects (typically 60%–100% incidence any morphological effect was acceptable) by 120 hpf, 2) RNA quality checks after RNA isolation, and 3) no more than two replicates for a given PFAS from the same day. A single control replicate was collected for each collection day, and control groups spread across all 96-well plates were required to exhibit less than 20% incidence any morphological effects. Exposure Round 2 consisted of a single collection day for PFAS 2, 4, 14, and 15, for which four replicates were selected for each chemical exposure group and for controls. Confirmation of EC_80_ values for these PFAS required more testing than others, necessitating separate exposures and collections from those in Round 1. In total, 70 samples, including 10 control replicates, were prepared for RNA-seq.

#### 2.4.3 RNA isolation and sequencing

Total RNA isolation was conducted using the Direct-zol RNA MiniPrep kit (Zymo, Cat no. R2052), and optional DNase I digestion step was performed. RNA concentration was measured using a SynergyMix microplate reader and Gen5 Take3 module (BioTek Instruments). Quality of the RNA was checked with an Agilent Bioanalyzer 2,100 at Oregon State University’s Center for Quantitative Life Sciences (CQLS), with all RIN ≥ 8.3. Samples underwent Lexogen QuantSeq 3′ mRNA-seq FWD library preparation and mRNA sequencing at the CQLS. All 70 samples were split between two P3 flow cells (each 1.2 billion reads per run) so that all individual samples were on both flow cells, and samples were sequenced using an Illumina NextSeq 2000 (100 bp single end). Sequenced read counts for individual samples in one flow cell ranged from approximately 5.5–13 million.

#### 2.4.4 RNA-seq data analysis

The quality of sequencing output was checked by inputing fastq files into FastQC (Andrews, 2015), and all samples were good quality with mean quality scores greater than 30. Two fastq files for each of the 70 samples (i.e., one fastq from each of the two flow cells between which each individual sample was split), 140 files in total, were processed separately during alignment. To achieve transcript counts, fastq files were aligned to the GRCz11 zebrafish genome using the STAR alignment tool (parameters: –outFilterType BySJout –outFilterMultimapNmax 20 –alignSJoverhangMin 8 –alignSJDBoverhangMin 1 –outFilterMismatchNmax 999 –outFilterMismatchNoverLmax 0.6 –alignIntronMin 20 ––alignIntronMax 1,000,000 ––alignMatesGapMax 1,000,000 ––outSAMattributes NH HI NM MD ––outSAMtype BAM SortedByCoordinate) (Dobin et al., 2013). During alignment of all fastq files, the percentage of uniquely mapped reads was >80%, except for two with approximately 75%. Gene-level counts were determined using HTSeq (parameters: m intersection-nonempty -s yes -f bam -r pos) (Anders et al., 2015). Subsequent curation and analysis was performed using R Statistical Software (v4.1.3) (Team, 2021). To remove genes with low counts, gene count files comprised of a total of 32,521 genes underwent filtering. Genes with zero counts for all samples were removed from the dataset (9.9%). Of the remaining genes, those that were in the lowest 35th percentile of counts for all files were removed (4.8%). Multidimensional scaling analysis in R (metaMDS function, vegan package) identified one outlier, a replicate for PFAS 5, which was removed prior to further analysis (Jari Okasen et al., 2022). Thus, all PFAS exposure groups were represented by four replicates, except for PFAS 5 with three replicates. Separate gene counts for each sample from the two flow cell runs were collapsed using the collapseReplicates function in DESeq2, and then normalization and differential expression analysis were performed using DESeq2 (Love et al., 2014). PFAS were compared to their respective controls based on Exposure Round (Figure 1). Genes were considered differentially expressed if they had an adjusted *p*-value < 0.05, with no fold change threshold to facilitate a more liberal, exploratory approach. Analysis of differentially expressed genes (DEG) and visualizations were conducted using a range of tools. PCA analysis was performed using the plotPCA function within DESeq2 with variance stabilized dataset as input. Overlap between DEG lists was investigated using InteractiVenn (Heberle et al., 2015), also used to generate venn diagram figures. Heatmaps were generated in R (heatmap.2, aheatmap, and ggplot function; ggplots and ggplot2 packages) with Euclidean distance function and ward.D2 clustering parameters (Warnes et al., 2016; Wickham, 2016). Functional enrichment analysis was conducted using g:Profiler under default settings, with data sources including gene ontology (GO), biological pathways (e.g., KEGG and Reactome), regulatory motifs in DNA, protein databases, and human phenotype ontology (Raudvere et al., 2019).

#### 2.4.5 Transcriptomic investigation of Nafion byproduct 2 (NB2) across developmental stages

To further explore findings from the initial transcriptomic portion of the present study, namely, no DEGs (48 hpf) despite morphological effects (120 hpf) after NB2 exposure, a follow-up transcriptomic experiment was conducted for NB2 with daily sampling across multiple timepoints, still at the single EC_80_ concentration. A separate series of exposures was conducted to obtain a new EC_80_ of 73 µM by 120 hpf for a newly purchased stock of NB2 (source and lot number unchanged from previous developmental toxicity and transcriptomic exposures). All exposure, sample collection, RNA isolation, and sequencing parameters were consistent with previously described Sections 2.4.1–2.4.4 except, sample collections were performed at 48, 72, and 96 hpf after embryos were exposed to NB2. Sampling over several days enabled probing of the onset of transcriptional changes across development. Alignment of data using STAR, HTSeq, and normalization and differential expression analysis using DESeq2 were also the same as in Section 2.4.4.

### 2.5 Body burden measurement of 5 select PFAS

#### 2.5.1 Chemical exposures

Investigation into the relationship between number of DEGs identified in the initial transcriptomics portion of this study and internal chemical concentration was performed in a follow-up experiment focused on the body burden of 5 PFAS still at their respective EC_80_ but across developmental stages. Embryos were exposed beginning at 6 hpf to 24 µM NB2 (PFAS 2), 16 µM PFOS (PFAS 3), 25 µM PFHpS (PFAS 5), 2.54 µM FOSA (PFAS 10), 6 µM FHxSA (PFAS 11), or 0.5% methanol as a control group, using the same paradigm as for the NB2 developmental toxicity section of this study (2.3.1). Chemicals were selected to represent diversity in number of DEGs and structural homology for comparison purposes, as well as chemical and analytical standard availability. Chemical concentrations were selected to achieve target percent incidence of approximately 80% effect to facilitate comparison to transcriptomics experiments (slightly different from previous confirmed EC_80_ values likely due to different stock solutions). As done for the transcriptomics sections of this study, hold back plates consisting of all chemical exposures (typically *n* = 16) were maintained until 120 hpf to ensure target percent effect was achieved. Exposures for all chemicals for body burden measurements were performed primarily across the span of 2 days, with an additional day performed for FOSA and FHxSA (Supplementary Data S1); all exposure paradigms were identical with controls on all plates and holdback plates maintained for each subset to ensure integrity and comparability of exposures.

#### 2.5.2 Sampling

Sampling for body burden measurements was conducted at 24, 48, 72, 96, and 120 hpf. Selected sampling timepoints facilitated comparison between chemicals at the 48 hpf timepoint, previously used for transcriptomics, and across developmental stages for a given chemical. Prior to sampling, external chemicals were washed from the fish within the 96-well plates by removing 50 µL exposure solution and conducting a series of additions and removals of 200 µL clean EM until sufficiently diluted (Rericha et al., 2022). At each timepoint, three replicates of 18–20 fish from each chemical group, and controls, were collected in safe-lock tubes. In addition to pooling fish within a chemical group, additional pooling between chemical groups was done to optimize analytical resources-individual replicates from PFOS, PFHpS, and NB2 exposures were pooled into 3 Group A replicates, and those from FOSA and FHxSA exposures were pooled into 3 Group B replicates. Separate groups were necessary as zebrafish have the capacity to metabolize sulfonamides into sulfonates (i.e., FOSA into PFOS) (Han et al., 2021). Once collected, fish were euthanized on ice, excess water was removed from tubes, and samples were homogenized with 200 µL PFAS-free water and 0.5 mm stainless steel beads in a Bullet Blender (Next Advance) at speed 8 for 6 min. Homogenates were stored at −20°C until processing for analytical measurement.

#### 2.5.3 Analytical measurements

Samples were prepared using a previously published method (Rericha et al., 2022) with minor modifications, all described in section S1. Briefly, during sample extraction, embryonic zebrafish homogenate was transferred into 15 mL PPE centrifuge tubes, spiked with 30 µL of surrogate standard and incubated for 10 min (Supplementary Table S2), acetonitrile was added, and samples underwent vortexing, sonication, and centrifugation. The liquids were decanted into new 15 mL centrifuge tubes, and the original tubes were rinsed with methanol. Ethylene glycol was added, and samples were evaporated then reconstituted in methanol. Sample cleanup was achieved using 250 mg Envi-carb tubes. Samples were decanted, evaporated, and reconstituted in 150 µL of methanol. Fifty µL aliquots were diluted with 40 µL of methanol, 50 µL of 2.3 mM NaCl, and 10 µL of internal standard.

Analytical-grade native (NBP2, PFOS, PFHPS, FOSA, and FHxSA) and mass-labelled standards (MPFHxS, MPFOS, M3PFBS, and M8FOSA) were purchased from Wellington Laboratories (Guelph, ON, Canada) (Supplementary Table S2). Quality assurance of the analytical process followed protocols described in section S1. All target PFAS were below the limit of detection (LOD) (Vial and Jardy, 1999) in all solvent blanks (Supplementary Table S3). Chemical analysis was performed using high-performance liquid chromatography (Agilent 1260 HPLC) with a SCIEX 5500 qTOF interface and an electrospray ionization source that was operated in a negative-ion mode as previously described (Schwichtenberg et al., 2020). Analytical measurement was achieved by LC-QToF as modified after Nilsen et al. (2024) (Nilsen et al., 2024). Briefly, analytical measurements were performed by LC-QTof using a Zorbax Eclipse C18 delay column (4.6 × 50 mm × 50 µm), an injection volume of 100 μL, and separation with an Eclipse C18 analytical column (4.6 × 75 mm × 3.5 µm), with more details in Supplementary Material.

#### 2.5.4 Body burden calculations

PFAS body burden in ng/mg body weight (ng/mg bw) was calculated by dividing the total measured mass for each PFAS in one sample by the number of fish collected in that sample from the exposure group of the chemical of interest, and then dividing by the average wet weight of a zebrafish at the appropriate timepoint. All data necessary for calculations provided in supplemental raw data spreadsheets. For example, considering a 48 hpf sample from Group A, consisting of 20 fish exposed to PFHpS, 20 fish exposed to PFOS, and 20 fish exposed to NB2 pooled together prior to the analytical analysis, where the total PFOS mass measured was 488 ng:

$$PFOS\;Body\;burden=\frac{488\;ng\;\left(total\;measured\;PFOS\;in\;sample\right)}{20\;fish\;\left(sampled\;from\;the\;PFOS\;exposures\right)\times 0.26\;mg\;\left(average\;body\;weight\;at\;48\;hpf\right)}$$

Several assumptions were made for these calculations, within each Group: 1) the total mass measured for a given PFAS in a sample was solely from the internal concentration in fish that were exposed to that PFAS, and 2) total mass measured was not the result of metabolism of other test compounds into target test compounds. Assumption 1 was likely valid based on 0 ng or <LOD PFAS concentrations measured in all control samples and measures taken to avoid PFAS contamination between separate PFAS exposures throughout the experiment. Assumption two was supported by published PFAS metabolism studies in zebrafish and the precaution taken to separate the sulfonamide PFAS from the sulfonate PFAS (Han et al., 2021). Internal concentrations were statistically compared between chemical treatments and timepoints using an ANOVA with Tukey’s post-hoc (adjusted *p*-value < 0.05).

## 3 Results

### 3.1 Nafion byproduct 2 (NB2) developmental toxicity

NB2 exposure (not previously tested by our laboratory with the 139 EPA PFAS library) induced developmental toxicity in the form of morphological and larval behavior effects. Significant incidence of craniofacial, muscular, and brain-region malformations, along with edema and abnormal touch response relative to controls was observed after exposure to 16 µM. After 35 μM, axis malformations were also noted (Supplementary Table S2). Overall, the calculated BMD_80_ for any morphological effect was 28.2 µM (Table 1; Supplementary Figure S1). NB2 did not affect embryonic behavior but did affect larval behavior. During the light period of the LPR assay, hypoactivity (i.e., decreased movement) occurred in response to 1.0 and 2.5 µM, which turned to hyperactivity at 6.5 µM. During the dark period of the assay, 1 and 6.5 µM exposures caused hypoactivity. At 16 µM and above, less than 40% of the exposed fish were phenotypically normal and behavior analysis was thus statistically invalid. The bioactivity of NB2 warranted its inclusion for the present transcriptomic investigation.

### 3.2 EC_80_ confirmation and phenotypic anchoring for 15 PFAS

During confirmation testing, EC_80_s for all but three PFAS (12, 14, and 15) were confirmed and utilized as the exposure concentrations for transcriptomics (Table 1). PFAS 14 and 15, the two diacrylate compounds, induced high incidence of mortality at 24 hpf, as was also observed during initial testing of the EPA PFAS library where the compounds elicited 86% and 64% mortality at 24 hpf, respectively, after 33.3 µM exposures, and both caused 100% 24 hpf mortality after 66.5 µM exposures (Truong et al., 2022). High mortality by 24 hpf drove the response profile and precluded anchoring 48 hpf transcriptomic data to teratogenicity at 120 hpf. Therefore, exposures to PFAS 14 and 15 for transcriptomics were conducted at 20 μM, a sub-teratogenic concentration. Exposure to PFAS 12 at 100 µM resulted in approximately 25%–64% incidence of morphological effects. Despite a lower-than-targeted response, exposures for PFAS 12 were performed at 100 µM and were still considered phenotypically anchored. Using 100 µM as a maximum concentration preserved limited chemical stock volume and avoided testing unreasonably high concentrations. Confirmation testing revealed that some EC_80_s were identical to previously modeled BMD_80_’s, though some were lower (PFAS 4) and some were higher (Table 1).

**FIGURE 2:**
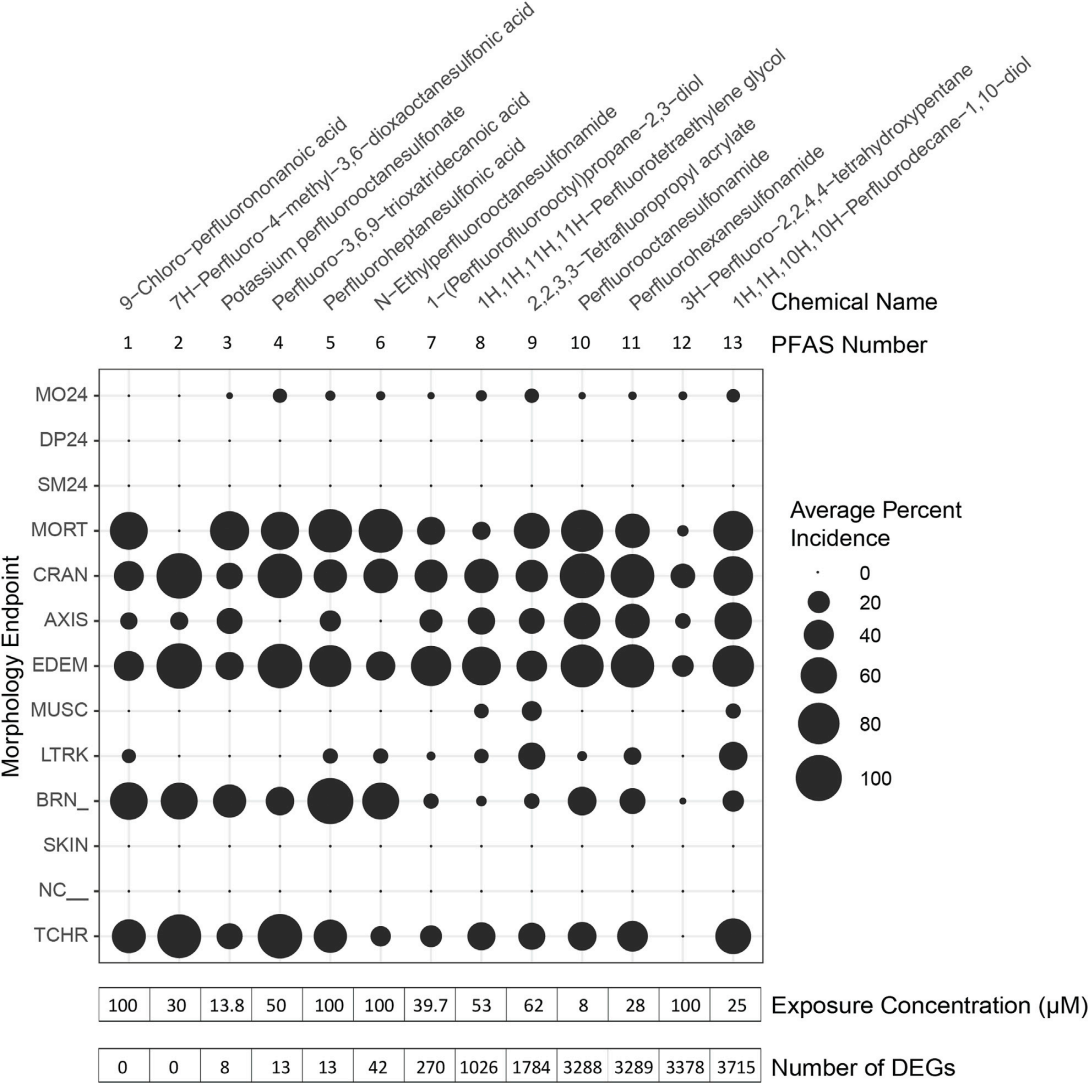
Morphological effects of PFAS exposures and phenotypically anchored transcriptomics. Zebrafish were exposed to PFAS at 6 h post fertilization (hpf), sampling for RNA sequencing performed at 48 hpf, and morphology assessments conducted at 24 and 120 hpf. PFAS for which phenotypic anchoring of transcriptomics was possible are named and numbered along the top of the figure. PFAS are listed in order (left to right) of increasing number of differentially expressed genes (DEGs), defined only by an adjusted *p*-value threshold of <0.05 and noted at the bottom of the figure. Average Percent Incidence of each morphological effect (*y*-axis) are displayed within the bubble plot, with larger bubbles signifying higher percent incidence. See Supplementary Table S1A and Supplementary Table S1B for descriptions of each morphological endpoint.

Despite some shifts between preliminary BMD_80_ and confirmed EC_80_, phenotypic anchoring of transcriptomics was achieved for PFAS 1–13. Following exposures at 6 hpf, PFAS 1–13 induced minimal mortality by 24 hpf but had induced significant teratogenicity by 120 hpf (Figure 2). The most severely affected endpoints included mortality, craniofacial and axis malformations, edemas, brain-region malformations, and abnormal touch response. The average incidence of any cumulative morphological effect at 120 hpf among the replicate days for all PFAS ranged from 77% to 100% after exposures to PFAS 1–13, except for 32% caused by PFAS 12 (Supplementary Table S5). Phenotypic anchoring for all but two of the tested PFAS facilitated investigation of early transcriptional changes, presumably closer to the molecular initiation of toxicity.

### 3.3 Differential gene expression for 15 PFAS

Gene expression profiles induced by 7 of the PFAS exposures were very similar to control profiles, as shown by similar clustering in principal component analysis (PCA), while other PFAS drastically altered gene expression (Supplementary Figure S2). Exposure to the early mortality inducing, and thus the non-phenotypically anchored, PFAS 14 and 15 did not elicit differentially expressed genes. Phenotypically anchored PFAS exposures (PFAS 1–13) resulted in a range of 0–3,715 DEGs (adjusted *p*-value < 0.05; Figure 2, where PFAS id number corresponds to the increasing number of DEGs and is the same as Table 1). To investigate whether lack of or fewer DEGs following exposures to PFAS 1–6 could be attributed to differences in morphological responses compared to PFAS 7–13, percent incidence of individual morphological endpoints was plotted in Figure 2. Some higher incidences of brain region malformations (“BRN_” in Figure 2) were observed for PFAS 1–6. However, no obvious trends between morphology endpoints and number of DEGs was observed.

PFAS 8–13 induced more than 1,000 DEGs each (Figure 2). Exposure to PFAS 13 (1H,1H,10H,10H-Perfluorodecane-1,10-diol) caused the most transcriptional changes with 3,715 DEGs, 1,696 of which increased in expression relative to controls and 2,019 decreased (Supplementary Table S6). Of the total 7,882 DEGs among PFAS 8–13, 192 DEGs were common among all six chemicals (Supplementary Figure S3; Supplementary Data S1). Large commonalities were also shared between PFAS 12 and 13 (219 DEGs), PFAS 10 and 12 (251 DEGs), PFAS 10, 11, and 13 (256 DEGs), and PFAS 11 and 13 (285 DEGs). PFAS 10 and 11, both consisting of sulfonamide functional head groups, induced many DEGs (3,288 and 3,289, respectively) and uniquely shared 298 DEGs. In addition to overlapping gene lists, the number of distinctive DEGs for each PFAS was also striking (Supplementary Figure S3). The percentages of unique DEGs compared to the total DEGs for each chemical were 7.3, 11, 30, 14, 36, and 21% for PFAS 8–13, respectively.

**FIGURE 3:**
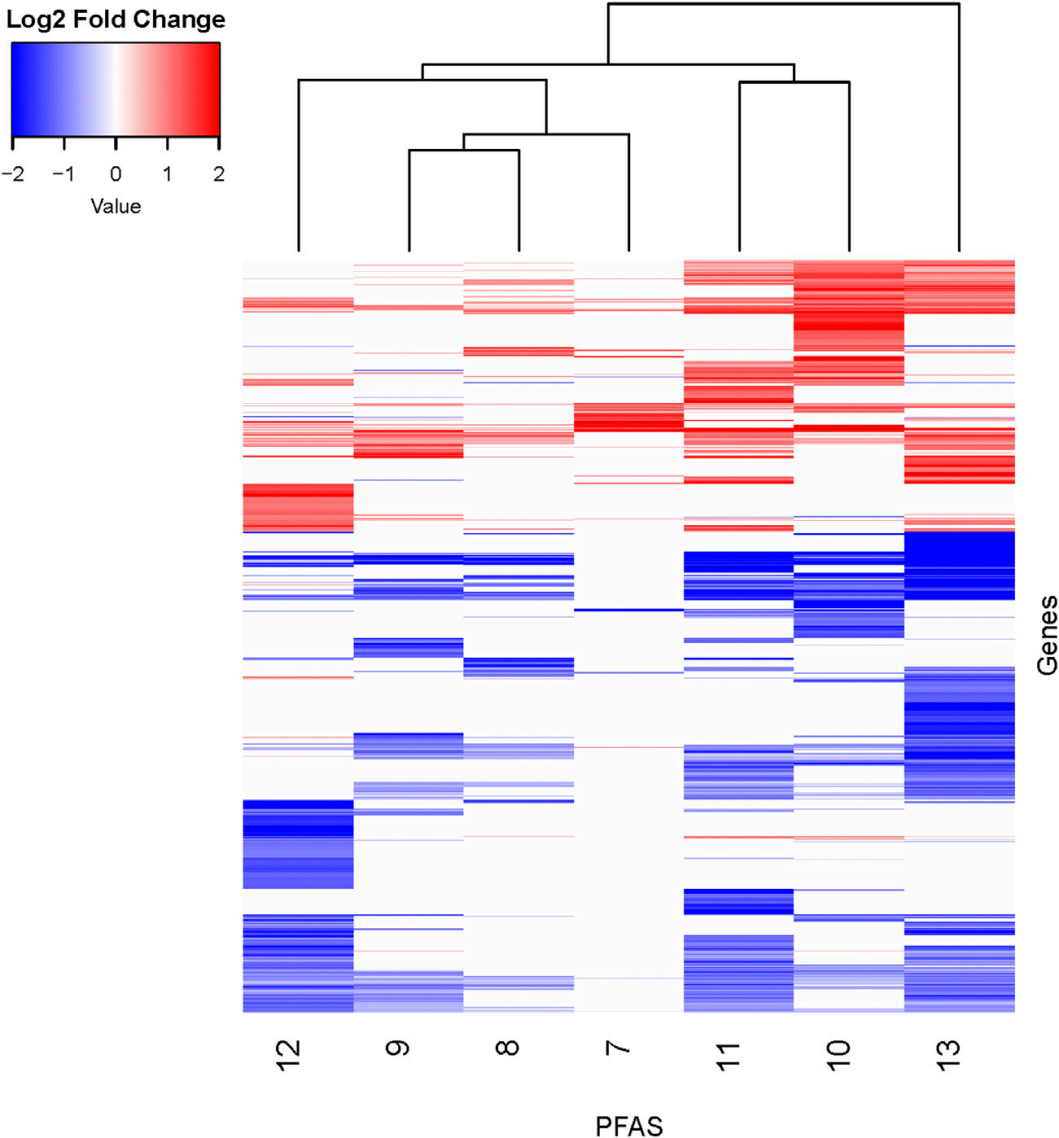
Heatmap of DEGs with additional fold change threshold applied. DEGs (padj < 0.05) for all PFAS underwent an additional filtering step of log_2_ fold change >|1| for the sake of visualizing similarities between compounds. PFAS with less than 10 DEGs remaining were not shown. PFAS 7–13 are noted at the bottom of the figure. Genes with transcripts that were increased in abundance based on log2 fold change compared to respective controls are red, and those with decreased abundance are blue. See Supplementary Table S6 for gene counts. Clustering analyses of the chemicals (top of figure) and genes (not shown) were performed.

PFAS exposures led to both similarities and differences in gene expression profiles. To further explore and visualize the data, a log_2_ fold change threshold of >|1| was applied, PFAS with less than 10 DEGs were removed, and clustering analyses performed (Figure 3; Supplementary Table S6). The structurally similar PFAS 10 (perfluorooctane sulfonamide) and 11 (perfluorohexane sulfonamide) clustered together based on the log_2_ fold change data shown in Figure 3. PFAS 7, 8, and 9 also clustered together, though they do not share overtly similar chemical structures. The gene expression changes produced by exposure to PFAS 7 (1-(Perfluorofluorooctyl) propane-2,3-diol) are noteworthy due to a lack of genes with decreased expression, though this subset of data was relatively sparse after implementing the fold change threshold. Alternatively, PFAS 13 exposure caused decreased expression of many DEGs above the log_2_ fold change threshold. Despite clustering and similarities based on hierarchical clustering of log_2_ fold change expression data, there are still distinctions between the expression profiles, which is also apparent based on clustering in PCA analyses of all normalized read counts (Supplementary Figure S2). It should be noted that hierarchical clustering was inherently affected by the total number of DEGs available for analysis for each compound after filtering. Overall, transcriptional responses to PFAS exposures shared both similarities and also unique differences that we further explored through functional enrichment analyses.

### 3.4 Functional enrichment analysis for 15 PFAS

**FIGURE 4:**
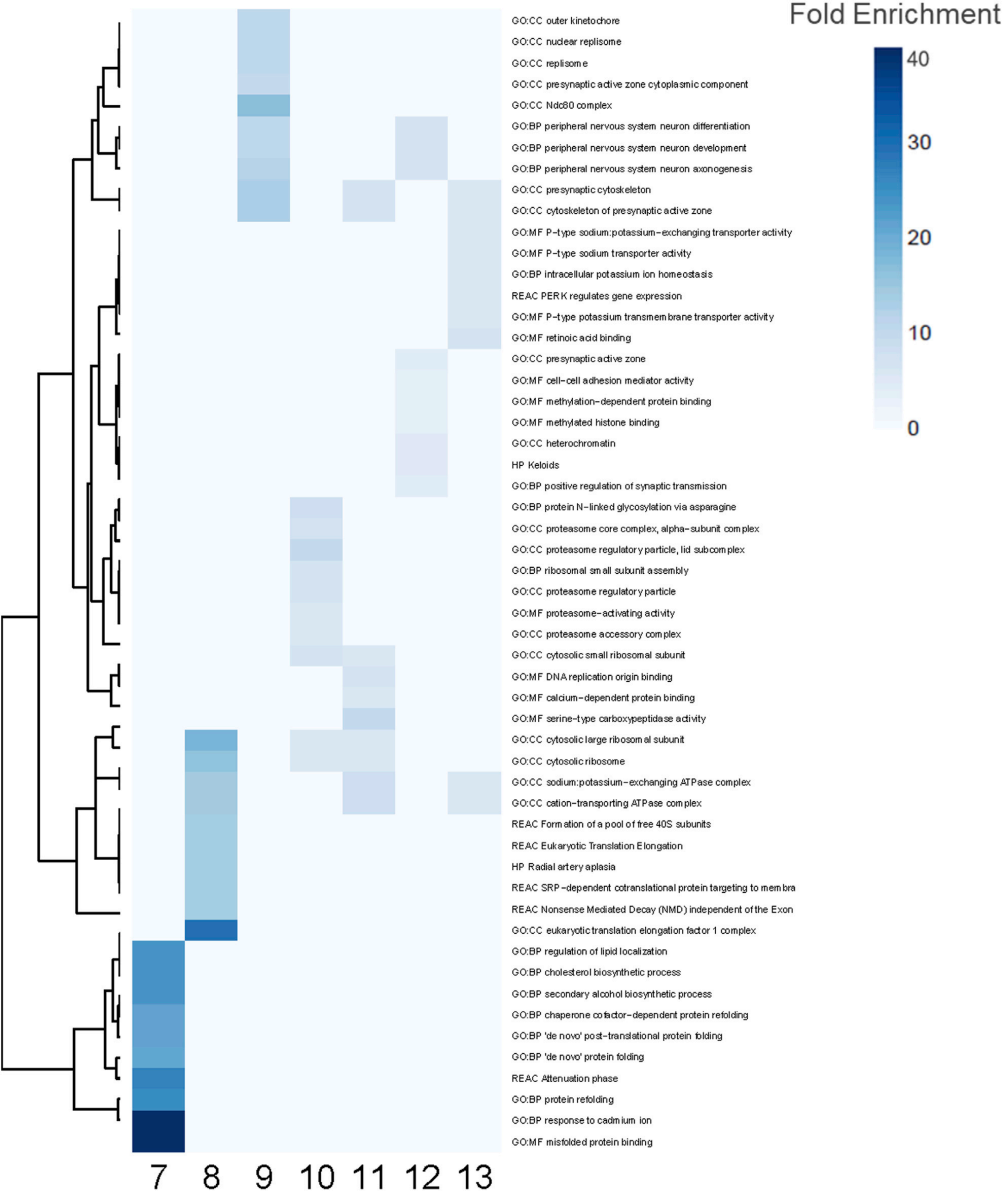
Enriched functional terms for PFAS 7–13. The top 10 most enriched functional terms were identified for each PFAS 7–13 and combined for a total of 54 processes. Blue indicates significant enrichment of a process by a given PFAS. Darker blue signifies higher fold enrichment, i.e., a higher percentage of DEGs associated with the process were dysregulated in the PFAS datasets.

Functional enrichment analyses similarly revealed common and unique biological processes perturbed by PFAS exposures (Figure 4). PFAS 1–6 did not elicit enough DEGs (adjusted *p*-value < 0.05) to enable robust functional enrichment analysis, therefore, only PFAS 7–13 were analyzed (Supplementary Data S1). The top ten most highly enriched functional terms for each PFAS were identified (Figure 4). Numerous PFAS shared their most highly enriched biological processes, including sodium potassium-exchanging ATPase complex and cation-transporting ATPase complex (PFAS 8, 11, 13), presynaptic cytoskeleton and cytoskeleton of presynaptic active zone (9, 11, 13), and cytosolic ribosome and cytosolic large ribosomal subunit (8, 10, 11). PFAS 9 and 12 exposures both elicited highly enriched terms related to peripheral nervous system neuron differentiation, development, and axonogenesis processes.

Among the 54 functional enrichment terms consisting of the top 10 most enriched among PFAS 7–13, many processes were also unique to individual PFAS (Figure 4). PFAS 10 uniquely induced processes related to proteasome regulatory particle and proteasome-activating activity among its most highly enriched. Within this subset of the most enriched functional terms, PFAS 7 notably did not share any with the other PFAS, and the same was true when considering the most significantly enriched (lowest adjusted *p*-value). Functional terms enriched by exposure to PFAS 7 included several related to protein folding (e.g., “*de novo*” protein folding, misfolded protein binding, and protein refolding), regulation of lipid localization, and cholesterol biosynthetic processes (Figure 4; Supplementary Figure S4). Among other PFAS, many terms related to general development (e.g., system development, multicellular organ development, developmental processes, and anatomical structure development) were among the most significantly enriched.

**FIGURE 5:**
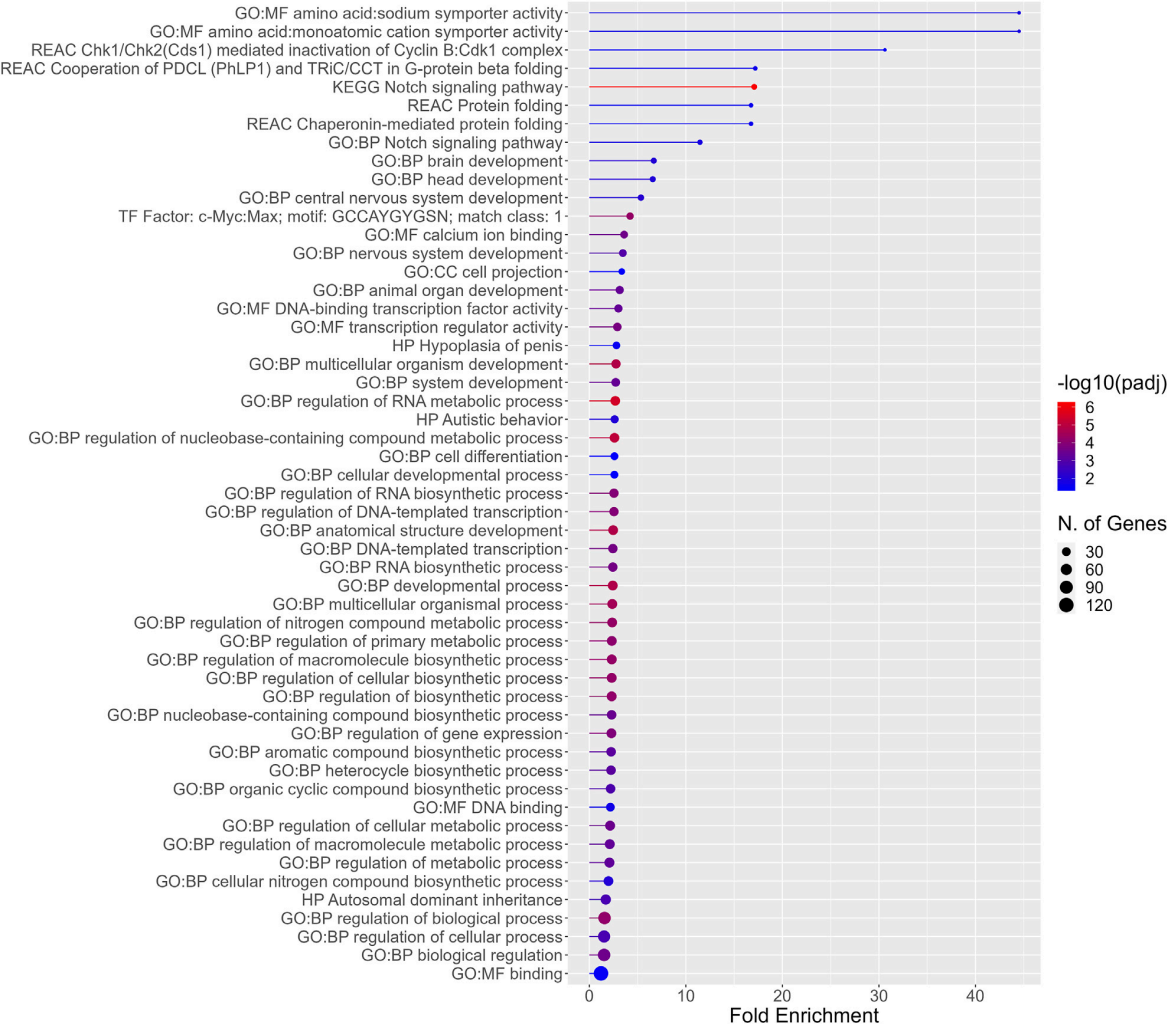
Functional enrichment for the 192 DEGs common among all PFAS 8–13. Higher fold enrichment is represented by bars that extend farther across the *x*-axis, a larger number of DEGS from the present dataset associated with a given process is signified by a larger dot (N. of Genes), and increasing significance of the enrichment is shown as the color of the dots approaches red (−log10padj).

In addition to functional enrichment analysis for the entirety of PFAS 7–13 DEGS, a separate analysis was also conducted for the 192 DEGs common to those PFAS that induced >1,000 DEGS (PFAS 8–13). The most highly enriched functional profiles included terms related to amino acid:monoatomic cation symporter activity, the notch signaling pathway, protein folding, and brain and nervous system development (Figure 5). Enriched processes also included regulation of RNA metabolic and biosynthetic processes, an assortment of metabolic processes, and multicellular organism development and anatomical structure development.

### 3.5 Body burden across developmental stages for 5 select PFAS

Body burden was measured for NB2, PFOS, PFHpS, FOSA, and FHxSA across a 24–120 hpf developmental time series to investigate whether internal concentration would provide insight into the range of DEGs observed at 48 hpf. Chemical selection provided some variation in functional head group (i.e., sulfonic acid and sulfonamide) and fluorinated carbon chain length. As previously stated, the selected PFAS induced 0, 8, 13, 3,288, and 3,289 DEGs (adjusted *p*-value < 0.05), respectively, and all induced morphological effects by 120 hpf. The body burden exposures were conducted with different chemical stocks (in methanol), and therefore also at slightly different concentrations, than used for transcriptomics (Supplementary Table S8). Hold back plates from exposures for body burden confirmed that all chemicals induced 63%–100% any morphological effect (Supplementary Data S1), meaning the same phenotypic anchoring approach that we applied to transcriptomic sampling was used.

**FIGURE 6:**
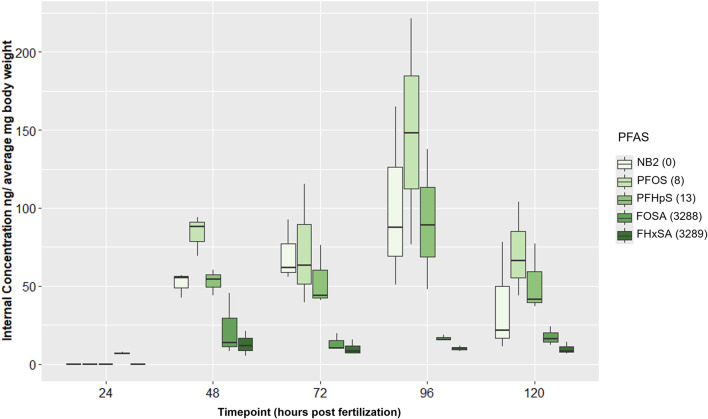
Internal concentrations (ng/average mg body weight) for 24 µM Nafion byproduct 2 (NB2; PFAS 2), 16 µM PFOS (PFAS 3), 25 µM PFHpS (PFAS 5), 2.54 µM FOSA (PFAS 10), and 6 µM FHxSA (PFAS 11) exposures across 24–120 hpf. PFAS are listed in order (left to right within each timepoint grouping) of increasing number of differentially expressed genes (DEGs) at 48 hpf. DEGs counts are noted to the right of each PFAS in the legend and were defined only by an adjusted *p*-value threshold of <0.05. Sulfonic acid PFAS (PFAS 2, PFAS 3, PFAS 5) ranged from 52–85 ng/mg compared to 13–22 ng/mg for the sulfonamide PFAS (PFAS 10, PFAS 11).

Trends in internal PFAS concentrations did not overtly correspond to trends in numbers of DEGs at 48 hpf. In all control samples, all PFAS were measured at 0 or <LOD, and no contamination between Group A and Group B chemical samples was detected. Internal concentrations for all PFAS were <LOD or below the limit of quantification (LOQ) at 24 hpf except FOSA (Supplementary Table S7), which averaged 7 ng/mg bw (Supplementary Table S8). Average internal concentrations of PFAS with a sulfonic acid head group (NB2, PFOS, and PFHpS) were greater than those of PFAS with a sulfonamide head group (FOSA and FHxSA) at 48 hpf and for the remainder of the time series (Figure 6), despite the two sulfonamide PFAS inducing far more DEGs at 48 hpf. Concentrations of the three sulfonic acid PFAS significantly increased from 24 to 96 hpf, and at 96 hpf, NB2 and PFOS concentrations were both significantly higher than FOSA and FHxSA (adjusted *p*-value < 0.05; Supplementary Table S9). Neither of the sulfonamide PFAS showed significant changes in internal concentration during the 24–96 hpf period. The highest average internal concentration was measured after the PFOS exposure at 96 hpf (150 ng/mg bw). Average internal concentrations for the sulfonic acid PFAS decreased from 96 to 120 hpf (though not statistically significantly); this decreasing trend may correspond to high incidence of malformations at 120 hpf. Despite random sampling and pooling of fish, high variation among replicate samples was observed and may be attributed to variation in uptake and morphological effects between individuals. Given the increasing internal concentration of the sulfonic acid PFAS between 24–96 hpf, we next investigated the transcriptional response to NB2 across a time series.

### 3.6 Differential gene expression across developmental stages for Nafion byproduct 2 (NB2)

**FIGURE 7:**
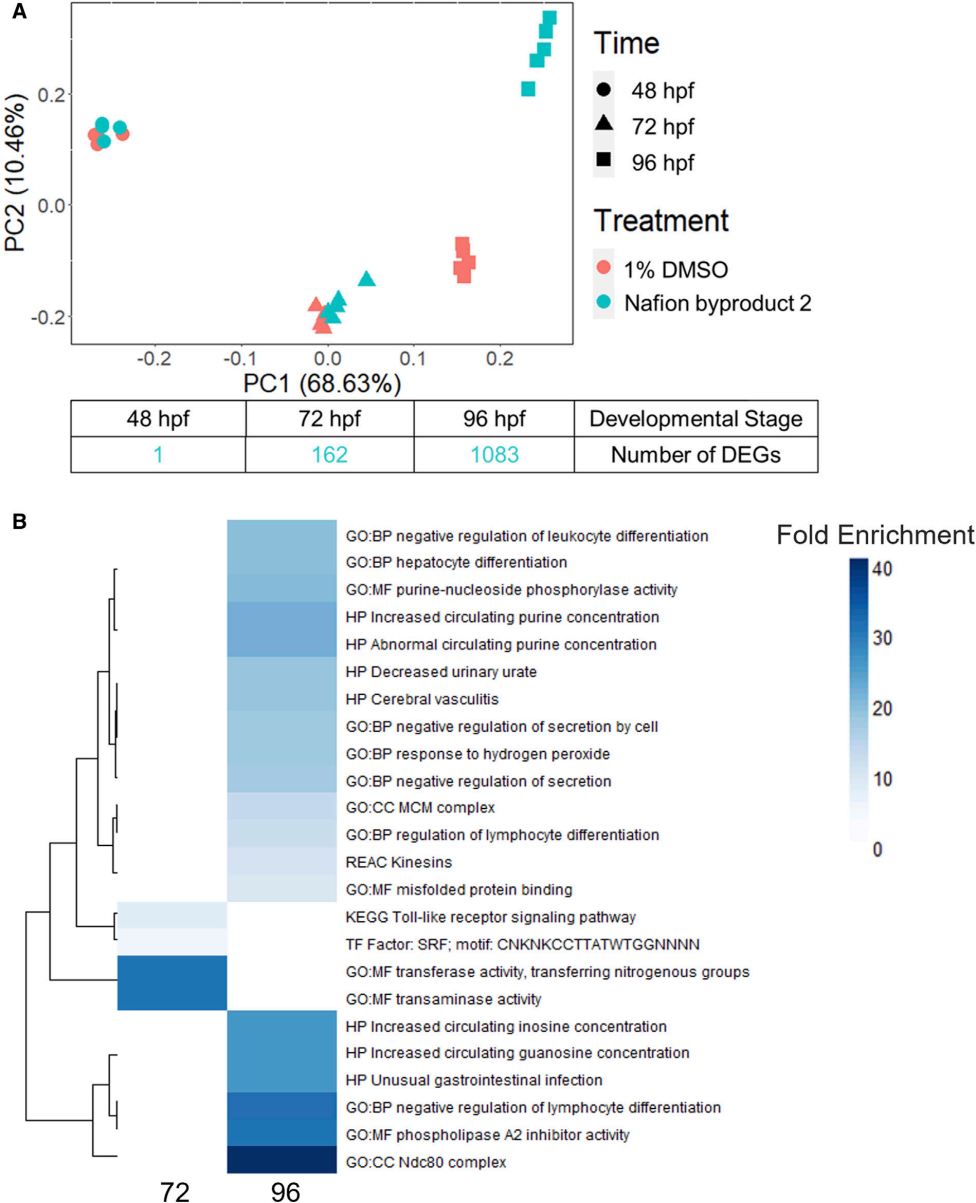
**(A)** A principal component analysis (PCA) plot of the top 10,000 most highly expressed genes across developmental stage (hours post fertilization) and treatment (control or 73 µM Nafion byproduct 2) as well as the number of differentially expressed genes (DEGs) identified at 48, 72, and 96 hpf. **(B)** The top 20 most enriched functional terms identified for Nafion byproduct two at the 72 and 96 hpf developmental stages. Darker blue indicates higher fold enrichment, although there were overlapping DEGs, significantly enriched functional terms were distinct between 72 and 96 hpf timepoints. There were four terms enriched at 72 hpf.

NB2 (also known as 7H-Perfluoro-4-methyl-3,6-dioxaoctanesulfonic acid) exposed animals produced significant morphological effects at 120 hpf, but 0 DEGs were identified at 48 hpf. To further investigate the NB2-specific molecular response in the zebrafish, we repeated developmental toxicity exposures with a new stock and collected animals across developmental stages (48, 72, and 96 hpf). Animals were severely malformed by 120 hpf and thus not appropriate for sampling as we were specifically targeting time points that preceded malformations. The EC_80_ achieved by the new NB2 exposures was 73 μM, at which 83% of animals exhibited morphological effects (Supplementary Figure S5). Gene expression profiles induced by NB2 across developmental stages were highly similar to controls until 96 hpf, as shown by similar clustering in the PCA plot in Figure 7A. At 96 hpf, the NB2 gene expression profiles diverged from controls and the number of DEGs increased from 162 at 72 hpf to 1,083 at 96 hpf. There were 66 overlapping DEGs between the 72 and 96 hpf time points. Following functional enrichment analysis, the most highly enriched terms at 72 hpf were related to transaminase and transferase activity, and processes related to kinetochore complexes (ndc80) and regulation of lymphocyte differentiation at 96 hpf (Figure 7B). The 66 overlapping DEGs between the 72 and 96 hpf timepoints showed the highest functional enrichment for transaminase and transferase activity (Supplementary Data S1).

## 4 Discussion

While PFAS toxicity testing has increased over the past decades, there is still limited understanding of the modes of action underlying PFAS toxicity. Further characterization of PFAS modes of action is essential to 1) begin to elucidate underlying mechanisms of toxicity, 2) inform PFAS grouping schemes and toxicity prediction tools, and 3) facilitate development of adverse outcome pathways (AOPs). To address this, the present study conducted a comparative transcriptomic investigation of 15 bioactive PFAS at a single developmental timepoint (48 hpf), followed by body burden measurements of 5 of the 15 PFAS and additional temporal transcriptomic assessment of one of the 15 PFAS, NB2, across developmental stages. The 15 selected PFAS represented many key structural features of PFAS. Compounds varied in functional head groups (e.g., carboxylic acid, sulfonic acid, sulfonamide, acrylate), number of head groups (e.g., diol, diacrylate), and fluorination (e.g., continuous fully fluorinated chains, polyfluorinated with ether groups). Due to the wide range of structures, these PFAS have varying physicochemical properties (outlined by Truong et al.) but all elicited toxicity in the developmental zebrafish model (Truong et al., 2022) with exposures aimed to achieve approximately 80% any morphological effect.

NB2 was identified as bioactive and therefore investigated alongside the 14 selected PFAS from the EPA library of 139 compounds. Nominal EC_80_ values were 30 and 73 µM for subsequent sections of this study (separate stocks), compared to an EC_50_ of 118.5 µM (55 mg/L) in recent literature, also in developing zebrafish (Gui et al., 2023a). In the present study embryos were dechorionated, whereas Gui et al. conducted exposures *in ovo*, which may explain the difference between effective concentrations. The morphological effects of NB2 exposure, and lower potency relative to PFOS exposures, were similar to those identified in other recent studies (Gui et al., 2023a; Phelps et al., 2023). Additionally, abnormal behavior caused by NB2 exposures as low as 1.0 µM indicated organismal-level effects even at lower, sub-teratogenic concentrations. Given its emergence as a contaminant of interest in recent years, combined with its bioactivity, evaluating NB2 developmental toxicity in the standardized system used to test the EPA PFAS library was important and added to the structural diversity of the present transcriptomic investigation (Kotlarz et al., 2020).

The morphological response profiles of two compounds, 1H,1H,5H,5H-Perfluoro-1,5-pentanediol (PFAS 14) and 1H,1H,6H,6H-Perfluorohexane-1,6-diol diacrylate (PFAS 15), both characterized by diacrylate functional groups and only differing by one -CF_2_- moiety (Table 1), were not conducive to phenotypic anchoring. These compounds induced high mortality by 24 hpf, before samples could be collected for transcriptomics at 48 hpf. The limited volume of stock remaining from the quantity obtained with the EPA library did not allow for extensive testing of these steep concentration-response curves. Within our paradigm, exposures to sub-teratogenic concentrations for both PFAS 14 and 15 did not yield differential gene expression compared to controls. Other studies, utilizing the same experimental paradigm, have also noted a lack of or low number of DEGs following chemical exposure to sub-teratogenic concentrations (Shankar et al., 2019; Rericha et al., 2022). Further testing of PFAS 14 and 15 and additional diacrylate PFAS, particularly focusing on toxicity and transcriptional response prior to 24 hpf, is warranted. Evidence of fluorotelomer acrylate compound biotransformation into potentially toxic intermediates in various models further highlights the need to better understand the biological lability and toxicity of structurally diverse PFAS with acrylate functional groups, particularly in developing organisms (Butt et al., 2010; Joudan et al., 2020). Despite challenges with these diacrylate compounds, phenotypic anchoring of early transcriptomic measurements was achieved for the remaining 13 PFAS.

Phenotypically anchored PFAS exposures (PFAS 1–13) resulted in a broad range of DEG counts, from 0 to 3,715 (adjusted *p*-value threshold of 0.05 with no fold change threshold). Despite relatively low DEG counts for several compounds, PFAS 7–13 elicited between 270 and 3,715 DEGs. One comparative approach to interpret the transcriptomic data was to contextualize trends in DEGs based on knowledge of chemical structures. Fluorinated chain length did not appear to influence the number of DEGs induced or the clustering of gene expression profiles (Figure 3). For instance, PFAS 1, 3, 6, 10, and 13 all have chains of 8 continuous perfluorinated carbons. The first three PFAS induced 0, 8, and 42 DEGs, whereas the second two induced over 3,000 DEGs each. On the other hand, similar gene expression clustering was apparent based on similarities in functional head group, particularly between sulfonate or sulfonamide PFAS when other structural features were also alike. PFAS 3 (PFOS) and 5 (PFHpS) both contain sulfonate head groups, with only one -CF_2_- moiety different between the two. Both compounds elicited an average of greater than 80% incidence any morphological effect at 120 hpf but caused only 8 and 13 DEGs at 48 hpf, with 6 DEGs common to both. PFAS 6, 10, and 11 have sulfonamide head groups, with 8, 8, and 6-fluorinated-carbon chains, respectively. PFAS 10 (FOSA) and 11 (FHxSA) both elicited over 3,000 DEGs and shared 31% of their cumulative DEGs, with highly similar patterns of increased and decreased gene expression (Figure 3). In a recent study also considering PFAS of a variety of structural categories, FHxSA exhibited the lowest transcriptomics-based point of departure compared to compounds such as PFOS in *Daphnia manga* (Villeneuve et al., 2024). Despite high structural similarity, PFAS 6 (n-ethyl perfluorooctane sulfonamide; EtFOSA) elicited far fewer DEGs (42) in the present study. Of PFAS 6’s DEGs, 35 were shared with either PFAS 10, PFAS 11, or both.

Presence of alcohol functional groups or identification as a polyether compound did not predict clustering based on gene expression, possibly due to various structural differences. PFAS 7 and 13, both diols with eight fluorinated carbons, did not cluster together based on gene expression profiles (Figure 3). Polyether compounds PFAS 2, 4, and 8 did not have similar numbers of DEGs that would enable comparisons. Finally, some compounds with relatively distinct structures, such as PFAS 8 (1H,1H,11H,11H-Perfluorotetraethylene glycol) and 9 (2,2,3,3-Tetrafluoropropyl acrylate), elicited similar DEG profiles based on clustering (Figure 3). Ultimately, some similarities between PFAS-induced gene expression were predictable based on PFAS structure, but no universal trends or sole driving structural features were identified.

Despite structural differences among the most transcriptionally disruptive PFAS (8–13), 192 DEGs were common among all exposures. These 192 DEGs were associated with functional enrichment terms related to brain and nervous system development, as well as general metabolic and biosynthetic processes. The 192 genes may be downstream transcriptional responses caused by the general disruption of development. Further investigation is needed to determine whether these common genes and associated biological processes are initiating drivers of toxicity or later key intermediate events leading to adverse outcomes. It is also possible that some transcriptional changes observed may have no functional or phenotypic consequence. Employing a phenotypically anchored paradigm for transcriptomics across a variety of compounds in a single, complex system, as done in this study, is an important step towards understanding biologically relevant transcriptional effects of PFAS exposures. However, further elucidation of mechanisms is essential to identify whether specific DEGs are players in initiating mechanisms and key events. Similarities, and also the many differences, in disrupted biological processes after PFAS exposures can provide insight into toxic modes of action at a higher level than individual genes and contribute to characterization among compounds.

Considering functional enrichment analysis across PFAS 7–13, similar terms were enriched following numerous PFAS exposures, though it is difficult to determine trends associated with PFAS structure. Processes related to sodium and potassium ion transport and homeostasis, as well as nervous and sensory system development (e.g., axon development, neuron projection development, eye morphogenesis, retina development in camera-type eye) were generally common for most of the PFAS exposures. Several PFAS altered genes associated with circulatory system and muscle development and cell differentiation, as well as erythrocyte differentiation and homeostasis (PFAS 8 and 10) and blood vessel and vascular development (PFAS 11). Previous RNA-seq studies in developing zebrafish identified disruptions in lipid metabolism, cellular homeostasis, cardiac function and development, and immune functions (Martinez et al., 2019; Sant et al., 2019; Christou et al., 2020; Dasgupta et al., 2020; Lee et al., 2021; Chen et al., 2022; Rericha et al., 2022). To our knowledge, PFAS 3 (PFOS), PFAS 10 (FOSA), and PFAS 2 (NBS) are the PFAS from the present study that have been previously investigated using RNA-seq in zebrafish (Gui et al., 2023a; Rericha et al., 2023). PFAS 3 (PFOS) elicited 8 DEGs and did not enable functional enrichment analysis, despite having been associated with high transcriptional alterations and a range of disrupted biological pathways in other recent studies. While presently identified biological processes perturbed by PFAS exposures generally align with the literature, pathways overtly related to lipid synthesis, transport, and metabolism, immune response, and the endocrine system (Rericha et al., 2023) were not observed in our datasets, though PFAS 7 exposure did alter genes associated with regulation of lipid localization and cholesterol biosynthetic process. The absence of altered biological processes commonly associated with PFAS exposures may be a result of the developmental timepoint selected for transcriptomic sampling (48 hpf). For instance, the liver is frequently identified as a target of PFAS toxicity with specific disruptions targeting lipid metabolism (Wang et al., 2022); at 48 hpf the zebrafish liver is nearing completion of the budding phase of development (Field et al., 2003). It is possible that liver toxicity and lipid dysregulation following PFAS exposures may occur later in development as the liver enters its growth phase at 50 hpf (Field et al., 2003), though others observed dysregulated transcripts related to hepatotoxicity as early as 14 and 24 hpf in zebrafish exposed to FOSA (Dasgupta et al., 2020). Direct comparisons between studies were difficult owing to experimental differences, such as mRNA sampling timepoint. In addition to mRNA sampling as early as 14 hpf (Dasgupta et al., 2020), others sampled at 120 hpf, compared to 48 hpf in the present study. Challenges arising from extrapolation of data between exposure and collection paradigms demonstrate the importance of the present study, which investigated 15 PFAS in a single system. Overall, the lack of DEGs after PFOS exposure was not atypical based on our findings as a whole; in fact, we saw several examples of PFAS that induced significant morphological effects by 120 hpf but little to no differential gene expression at 48 hpf.

Two phenotypically anchored compounds (PFAS 1 and 2) elicited 0 DEGs, and several others (PFAS 3–6) induced only a small number of DEGs at 48 hpf. While lack of both morphological effects and transcriptomic response would be biologically reasonable, the occurrence of a morphological phenotype paired with a lack of transcriptional changes required further interpretation. In previous studies of other chemical classes, robust morphological effects (120 hpf) were typically preceded by significant transcriptional changes at 48 hpf (Shankar et al., 2019; Dasgupta et al., 2021). This phenotypic anchoring paradigm enables investigation of early transcriptional changes that may be closer to molecular initiating events leading to toxicity. However, this paradigm did not yield differential gene expression for several PFAS and therefore may not be ideal for some compounds of this chemical class. Potential explanations for the discordance between DEGs and morphological effects may be that some PFAS only reach an internal concentration at the threshold of toxicity later in development due to toxicokinetic factors, perhaps biological targets of PFAS toxicity not being expressed until later in development, or a combination of the two. To further investigate, interrogation of body burden and transcriptional response across developmental stages for select PFAS was performed.

Body burden measurements of PFAS 2, 3, 5, 10, and 11 (NB2, PFOS, PFHpS, FOSA, and FHxSA, respectively), with all exposures eliciting at least 63% incidence of any morphological effect, revealed no qualitative positive relationship between DEGs and higher internal chemical concentration by 48 hpf. PFAS 2, 3, and 5 (sulfonic acid functional groups) exposures at their target EC_80_s elicited 0, 8, and 13 DEGs, far fewer than the >3,000 induced by PFAS 10 and 11 (sulfonamide functional groups). However, the sulfonic acid PFAS were measured at higher internal concentrations at 48 hpf, ranging from 42–94 ng/mg bw, compared to the sulfonamides measured at 5.3–45 ng/mg bw (Figure 6). Considering these results, the sulfonamides FOSA and FHxSA are particularly potent inducers of transcriptional changes early in development, despite lower internal concentration compared to other selected compounds.

Considering average internal concentrations across developmental stages, those of the sulfonic acid PFAS 2, 3, and 5 continuously increased from 24 to 96 hpf, while the sulfonamide PFAS 10 and 11 were relatively lower and stable across time (Figure 6; Supplementary Table S9). Increasing sulfonic acid PFAS body burdens were supported by existing literature for zebrafish exposed *in ovo* to PFOS (Vogs et al., 2019), but the lower sulfonamide PFAS concentrations were not in alignment with previous literature. Han et al. identified perfluoro-3,6,9-trioxatridecanoic acid (PFAS 4), then FOSA (PFAS 10) and PFOS (PFAS 3) as particularly bioaccumulative with the highest bioconcentration factors (BCFs) of 74 PFAS tested. Present findings concur with the high bioaccumulation potential of PFOS compared to other PFAS. However, the relatively low internal concentrations of FOSA during this developmental period up to 120 hpf was unexpected, possibly the result of chemical-induced toxicity.

BCF values (steady state) were calculated for the present study (Supplementary Table S10) to enable precursory comparison to the literature and were lower than in several other studies (Menger et al., 2019; Vogs et al., 2019; Han et al., 2021), with several potential explanations. Firstly, nominal instead of measured exposure concentration was used for all calculations. Additionally, unlike other studies, we intentionally conducted exposures at relatively higher nominal concentrations shown to cause morphological effects to match phenotypic anchoring in transcriptomic experiments. Therefore, biological systems important for typical toxicokinetics may have been affected in the fish at 120 hpf and earlier timepoints, and direct BCF comparisons between studies may not be appropriate. Furthermore, higher concentrations have previously been shown to elicit lower BCF, and other experimental factors such as whether embryos remained in the chorion, the ratio of number of embryos to exposure medium, and duration of exposure (5 or 6 days) may greatly affect BCF values (Menger et al., 2019; Vogs et al., 2019). Finally, an important note is that exposures and toxicokinetics in developing zebrafish experiments typically do not represent steady state conditions, though many studies calculate a steady state BCF. Kinetic BCF (Satbhai et al., 2022) may be more appropriate but was not calculated presently because of the expectation of morphological effects in our experiment and to enable comparison to steady state BCF values from the literature. Overall, within our experimental paradigm, the most effective means to investigate trends between body burden and transcriptional changes over time was to focus on the measured internal concentrations.

Given increasing internal concentrations of sulfonic acid PFAS over time, phenotypic anchoring (120 hpf), and the lack of DEGs at 48 hpf, we hypothesized that significant transcriptional response would occur later in development, closer to the onset of adverse effects in the animals. To test this, the trajectory of transcriptional response across developmental stages for NB2, a polyether sulfonic acid PFAS, was investigated. While NB2 exposure induced minimal DEGs at 48 hpf, it led to robust transcriptional response by 96 hpf with 1,083 DEGs identified (Figure 7A). The increased number of DEGs from 48 to 96 hpf reflected the increasing trend in internal concentration of NB2 between 48 and 96 hpf. However, as the internal concentration lacked statistical significance (adjusted *p*-value: 0.77); Supplementary Table S9), the divergence in transcriptomic profiles at 96 hpf may also stem from the emergence of a developmental stage-specific biological target. The most enriched molecular function (MF) at 96 hpf was phospholipase A2 (PLA2) inhibitor activity, where PLA2 plays a role in both lipid metabolism and inflammation (Farber et al., 1999). Interestingly, the most enriched biological process (BP) was negative regulation of lymphocyte differentiation, and previous studies have shown that PFOS, another sulfonic acid PFAS, altered lymphocyte subpopulations where exposed rats showed lower leukocyte counts (Ehrlich et al., 2023). Other recent studies in developing zebrafish identified enriched biological pathways related to lipid homeostasis, such as triglyceride and neutral lipid catabolic and metabolic processes, intestinal homeostasis and more in 120 hpf zebrafish (Gui et al., 2023b). Additionally, transaminase activity showed the highest fold enrichment for the 66 overlapping DEGs between 72 and 96 h and is typically used as a marker of liver toxicity but plays a role in many different biological processes (Kobayashi et al., 2020). It is important to note that the functional disruptions identified for NB2 would have otherwise been missed if transcriptomic assessments were only performed at 48 hpf, prior to the onset of liver growth and other important physiological structures that possess potential biological targets.

The current study assessed phenotypically anchored transcriptomic response to a set of structurally diverse PFAS, many of which had not previously been investigated using RNA-seq in zebrafish. Conducting such a study in a single, standardized, *in vivo* model enabled direct comparison of altered gene expression and disrupted biological pathways between compounds in a complex system. Differences in gene expression profiles and disrupted biological processes suggested that PFAS caused toxicity on different time courses or potentially through differing modes of action. It was necessary for all transcriptomic measurements to be performed using whole embryo or larvae homogenates due to the small size of embryos. The lack of tissue-specific insight and the systemic nature of the resulting DEGs and functional enrichment analyses should be noted while interpreting this dataset. We also acknowledged that PFAS metabolism was not directly addressed in the present study. A couple of the PFAS selected for the body burden investigation are terminal degradation products (e.g., PFOS and PFHpS). However, sulfonamide PFAS can be metabolized into sulfonic acids in zebrafish (e.g., FOSA to PFOS, and EtFOSA to FOSA to PFOS) (Han et al., 2021), though any amounts of PFOS in the Group B (FOSA and FHxSA) body burden samples were below the LOD. In Group B, an increase of PFHxS, a potential metabolite of FHxSA, was observed over time. Concentrations of PFHxS rose above the LOQ to concentrations of 160,000–178,000 ng/L (3.9–4.3 ng/mg bw if assuming the source of PFHxS is solely from the twenty fish exposed to FHxSA) in two out of the three replicates, but only at 120 hpf. Internal chemical concentrations, including any potential metabolism, for samples used in the transcriptomic portions of this study were not assessed. Future studies should conduct transcriptomics at multiple developmental timepoints between 24 and 120 hpf while also measuring internal concentrations if possible. Conducting multi-omics investigations incorporating proteomics and metabolomics may provide further insight into modes of action and potential mechanisms of toxicity. As more ‘omics data is generated for structurally diverse PFAS, interpretation of findings into modes of action, adverse outcome pathway frameworks, and characterization among compounds will be possible.

## 5 Conclusion

Developmental exposures to structurally diverse PFAS in zebrafish and anchoring of the ensuing teratogenic responses to the underlying transcriptomic changes revealed a broad range of gene expression profiles. Phenotypically anchored PFAS exposures induced DEG counts ranging from 0 to 3,715. While there were similarities in transcriptional changes between PFAS, such as between FOSA and FHxSA, stark differences in DEGs were also apparent. Commonly perturbed biological processes across many of the PFAS were generally related to ion homeostasis, nervous, sensory, and circulatory system development, and muscle development, while some processes were unique to specific PFAS. Lack of DEGs at 48 hpf despite teratogenic effects at 120 hpf following some PFAS exposures, including NB2, PFOS, and PFHpS, may be partially explained by lower internal concentrations early in development that steadily increased through 96 hpf. NB2 transcription also increased from minimal DEGs at 48 hpf to 1,083 at 96 hpf. Our standardized paradigm for transcriptomic investigation of early molecular changes leading to organismal-level effects was well-suited to begin to elucidate modes of action between many PFAS, though some will require additional transcriptomics across developmental stages to better understand their toxicology.

## Data Availability

The datasets presented in this study can be found in online repositories. The names of the repository/repositories and accession number(s) can be found in the article/Supplementary Material.
