# Supplementary material for: Diverse PFAS produce unique transcriptomic changes linked to developmental toxicity in zebrafish
Source: Front Toxicol. 2024 Jul 22;6:1425537. doi: 10.3389/ftox.2024.1425537 (PMC11298493; doi:10.3389/ftox.2024.1425537)
Supplement: Supplementary file 1 [file DataSheet1.zip › Proofs and Raw Data FINAL Submission/Raw Data Guide.docx]

Spreadsheets and Tabs:

- **15_PFAS_Transcriptomics_holdback_plates_Morphology:** Raw data from hold back plates for the transcriptomic investigation of 15 PFAS.
  - Mortality & Morphology: See Data Type descriptions below.
  - Corresponding RNA-seq sampling: Spreadsheet of metadata for the samples used for the 15 PFAS RNA-sequencing, specifically which replicates were exposed on each day to enable mapping back to representative hold back plates by date of exposure.
- **15_PFAS_Normalized_Counts:** Normalized counts obtained from DESeq2 (variance stabilized) after data pre-processing and outlier removal.
- **15_PFAS_Differential_Gene_Expression_DEGs+gProfiler:** Differential expression analysis output from DESeq2 with each PFAS analyzed against their respective control based on Exposure Round described in the manuscript. This spreadsheet has results filtered to only include DEGs as defined by an adjusted p-value less than 0.05. Functional enrichment analysis output from gProfiler using default settings and inputting DEGs for PFAS with sufficient DEGs to enable the analysis (7-13).
  - One tab for each PFAS that elicited DEGs and the coinciding functional enrichment terms.
  - Final tab for the 192 shared DEGs among PFAS 8-13.
- **5_PFAS_Body_Bruden_Results_and_Calculations:** Raw data from hold back plates for the body burden investigation of PFOS, PFHpS, NB2, FOSA, and FHxSA. See Data Type descriptions below, with an additional metadata column (For.sampling.timepoint) that indicates for which sampling timepoint the hold back plate is representative. Internal concentration measurements and calculations for body burden investigation of PFOS, PFHpS, NB2, FOSA, and FHxSA with associated sampling data necessary to calculate ng PFAS/average mg bodyweight.
  - Measured- ng per L: Analytical measurements for internal concentration (ng/L).
  - Fish pooled per sample: Number of fish pooled per sample following Group A and Group B pooling described in the manuscript. This information was used to calculate ng PFAS per individual fish.
  - Average bodyweight at lifestage: Fish were pooled and weighed in replicates at all life stages assessed during body burden investigation (24-120 hours post fertilization). The average bodyweight (wet weight) for each life stage was used to calculate ng PFAS/average mg bodyweight.
  - Calculated- ng PFAS per sample: ng PFAS/sample (200µL volume) was calculated.
  - Calculated- ng PFAS per mg bw: ng PFAS/average mg bodyweight was calculated for each sample following the assumptions described in the manuscript.
- **Nafion_byproduct_2_morphology:** Raw data from the initial developmental toxicity assessment of Nafion byproduct 2 (NB2).
  - Mortality & Morphology: See Data Type descriptions below.
  - Behavior- EPR: Embryonic Photomotor Response Assay data. See Data Type descriptions below.
  - Behavior- LPR: Larval Photomotor Response Assay data. See Data Type descriptions below.
  - New NB2 Stock Mort & Morph: Raw data from the mortality and morphological screening done for new NB2 stocks meant for the developmental stage RNA-sequencing.
- **Nafion_byproduct_2_DEGs+gProfiler:** Differential expression analysis output from DESeq2 with nafion byproduct 2 (NB2) analyzed against the respective control from the NB2 developmental stage exposures described in the manuscript. Functional enrichment analysis output from gProfiler using default settings and inputting DEGs for NB2 developmental stages with sufficient DEGs to enable analysis.
  - Normalized_Gene_Exp_Levels: Normalized read counts output from DESeq2.
  - DEGs_Naf_v_DMSO_48hpf: 48 hpf results filtered to only include DEGs as defined by an adjusted p-value less than 0.05 compared to controls.
  - DEGs_Naf_v_DMSO_72hpf: 72 hpf results filtered to only include DEGs as defined by an adjusted p-value less than 0.05 compared to controls.
  - DEGs_Naf_v_DMSO_96hpf: 96 hpf results filtered to only include DEGs as defined by an adjusted p-value less than 0.05 compared to controls.
  - Common_DEGs_72_96hpf: The 67 shared DEGs between 72 and 96 hpf.
  - gProfiler_72hpf: Functional enrichment terms identified at the 72 hpf developmental stage.
  - gProfiler_96hpf: Functional enrichment terms identified at the 96 hpf developmental stage.
  - Overlapping_72_96_gProfiler: Overlapping functional enrichment terms between 72 and 96 hpf developmental stages.

Data Types:

***Mortality and Morphology****:* This is animal/well level data (binary for presence or absence of phenotype) where each row represents an animal. There are 19 total columns. The first 5 columns are metadata including chemical name (or PFAS number), nominal exposure concentration (µM), an id for each 96-well plate, location of each animal within a 96-well plate, and the date of fertilization for that animal. These are followed by 13 columns for mortality and morphology endpoints at either 24 or 120 hours post fertilization (hpf), and also a well quality control column (“DNC_”). Below in Table A is a description of each endpoint.

*Note: If an embryo is dead (noted as "MO24", all subsequent endpoints will be "NA" as only viable embryos are evaluated). Therefore, the MORT column represents only those that are dead at 120 hours post fertilization (hpf) and does not take into consideration those dead at 24 hpf. To consider total mortality, if MO24 == 1, MORT should be 1 also. Additionally, the endpoint “DNC_” is not a morphological endpoint, but a well quality control. Therefore, if DNC == 1, that well should be discarded from the analysis completely.*

***Behavior (EPR and LPR)****:* Each row in the behavior tabs represents one well (i.e., animal) over time. For each behavioral tab, the first 4 columns consist of metadata, including chemical name, nominal exposure concentration (µM), plate id, and location of well in the 96-well plate, followed by the time series data. For 24 hpf behavior (EPR), there are 50 data points (every second), and for 120 hpf behavior (LPR) there are 240 datapoints (every 6 seconds).

*Note: Removal of dead or malformed fish is high recommended, but all data provided is uncensored. The timepoints for 24 hpf behavior (EPR) are broken down into 3 periods: T1:29 is Background; T31:39 is Excitatory; and T41-T48 is Refractory. The light (L) and dark (D) cycles in the 120 hpf behavior assay (LPR) are as follows: L1: T61-89; D1: T90-119; L2: T120-149; D2: T150-179; L3: T180-209; D3: T210-239. The first 2 cycles was treated as acclimation and the third cycle was analyzed.*

**Table A.** Mortality and morphology endpoint descriptors.

| Column Names | Definition |
| --- | --- |
| conc | Concentration for each well (µM) |
| plate.id | Plate barcode ID |
| well | Well placement on plate |
| date_of_plate | Date of fertilization for the animal / date the plate was exposed |
| MO24 | Mortality observed at 24 hpf |
| DP24 | Delayed developmental by 24 hpf |
| SM24 | Spontaneous movement at 24 hpf |
| MORT | Mortality occurring between 24 and 120 hpf |
| CRAN | Malformed, missing or smaller than normal the eye, snout, and/or jaw at 120 hpf |
| AXIS | Curved or bent axis in either direction at 120 hpf |
| EDEM | Heart and/or yolk sac malformation, pericardial or yolk sac edema (fluid around the heart) at 120 hpf |
| MUSC | Lack of circulation, malformation or disorganized/ missing somites, and improper swim bladder formation at 120 hpf |
| LTRK | Malformation of the lower trunk, including caudal fin region at 120 hpf |
| BRN_ | Brain malformations or necrosis at 120 hpf |
| SKIN | Abnormal pigmentation at 120 hpf |
| NC__ | Notochord malformation at 120 hpf |
| TCHR | Not responsive to touch at 120 hpf |
| DNC_ | Well quality control: 1 = poor well quality or animal was sampled before evaluation |
